# Water Resources in Africa under Global Change: Monitoring Surface Waters from Space

**DOI:** 10.1007/s10712-022-09700-9

**Published:** 2022-04-20

**Authors:** Fabrice Papa, Jean-François Crétaux, Manuela Grippa, Elodie Robert, Mark Trigg, Raphael M. Tshimanga, Benjamin Kitambo, Adrien Paris, Andrew Carr, Ayan Santos Fleischmann, Mathilde de Fleury, Paul Gerard Gbetkom, Beatriz Calmettes, Stephane Calmant

**Affiliations:** 1 LEGOS, Université de Toulouse, IRD, CNES, CNRS, UPS, 31400 Toulouse, France; 2 Institute of Geosciences, Universidade de Brasília (UnB), 70910-900 Brasília, Brazil; 3 GET, Université de Toulouse, IRD, CNES, CNRS, UPS, 31400 Toulouse, France; 4 LETG, CNRS, Université de Nantes, 44312 Nantes, France; 5 School of Civil Engineering, University of Leeds, Leeds, LS2 9DY United Kingdom; 6 Congo Basin Water Resources Research Center (CRREBaC) and Department of Natural Resources Management, University of Kinshasa (UNIKIN), Kinshasa, Democratic Republic of the Congo; 7 Department of Geology, University of Lubumbashi (UNILU), Route Kasapa, Lubumbashi, Democratic Republic of the Congo; 8 Hydro Matters, 31460 Le Faget, France; 9 Hydraulic Research Institute (IPH), Federal University of Rio Grande do Sul (UFRGS), 91501-970 Porto Alegre, Brazil; 10 Instituto de Desenvolvimento Sustentável Mamirauá, 69553-225 Tefé, AM Brazil; 11 Collecte Localisation Satellites (CLS), 31520 Ramonville Saint-Agne, France; 12 Institute de Recherche pour le Développement (IRD), Cayenne IRD Center, 97323 French Guiana, France

**Keywords:** Africa, Hydrology, Surface water, Remote sensing, Modeling, Review

## Abstract

**Abstract:**

The African continent hosts some of the largest freshwater systems worldwide, characterized by a large distribution and variability of surface waters that play a key role in the water, energy and carbon cycles and are of major importance to the global climate and water resources. Freshwater availability in Africa has now become of major concern under the combined effect of climate change, environmental alterations and anthropogenic pressure. However, the hydrology of the African river basins remains one of the least studied worldwide and a better monitoring and understanding of the hydrological processes across the continent become fundamental. Earth Observation, that offers a cost-effective means for monitoring the terrestrial water cycle, plays a major role in supporting surface hydrology investigations. Remote sensing advances are therefore a game changer to develop comprehensive observing systems to monitor Africa’s land water and manage its water resources. Here, we review the achievements of more than three decades of advances using remote sensing to study surface waters in Africa, highlighting the current benefits and difficulties. We show how the availability of a large number of sensors and observations, coupled with models, offers new possibilities to monitor a continent with scarce gauged stations. In the context of upcoming satellite missions dedicated to surface hydrology, such as the Surface Water and Ocean Topography (SWOT), we discuss future opportunities and how the use of remote sensing could benefit scientific and societal applications, such as water resource management, flood risk prevention and environment monitoring under current global change.

**Article Highlights:**

- The hydrology of African surface water is of global importance, yet it remains poorly monitored and understood
- Comprehensive review of remote sensing and modeling advances to monitor Africa’s surface water and water resources
- Future opportunities with upcoming satellite missions and to translate scientific advances into societal applications

## Introduction

Freshwater on land is a vital resource for terrestrial life, ecosystems, biodiversity and human societies (Vörösmarty et al. 2000, 2010; Steffen et al. 2015; Seddon et al. 2016; Albert et al. 2021). Continental water is stored in various reservoirs, unevenly distributed across geophysical environments and climates (Chahine 1992; Shiklomanov and Rodda 2003). It includes seasonal ice and snow, glaciers and ice caps, aquifers, soil water and soil moisture, and surface waters (Stephens et al. 2020). The latter, comprising of rivers, lakes, man-made reservoirs, wetlands, floodplains and inundated areas (Alsdorf et al. 2007), is of particular significance as it supports diverse and dynamic environments globally and provides important benefits and services to human society and economic activities (Oki and Kanae 2006).

Surface waters are also an integral part of the global water cycle (Good et al. 2015; Trenberth et al. 2007), continuously exchanging mass with the atmosphere and the oceans, making them a key component of the climate system and its variability (Stephens et al. 2020).

However, freshwater storage and flux, their spatial distribution and variability, remain highly unknown in many regions of the world (Rodell et al. 2018), preventing the development of adequate and sustainable strategies to manage water resources (Oki and Kanae 2006; Hall et al. 2014).

This context leaves open major questions regarding the contemporary distribution of water availability across lands (Alsdorf et al. 2007): How much freshwater is stored across the surface of continents? What is the spatiotemporal dynamic of surface freshwater and how does it interact with climate variability and anthropogenic pressure?

These questions are of particular relevance for Africa, the second-largest continent in the world, both in size and population. The region occupies ~ 30 million km^2^ and hosts almost 1.4 billion inhabitants as of 2022, currently ~ 18% of the global population (United Nations 2019). Africa’s population is expected to double by 2050 with the share of global population projected to grow to 26% in 2050 and possibly ~ 40% by 2100 (United Nations 2019).

**Fig. 1:**
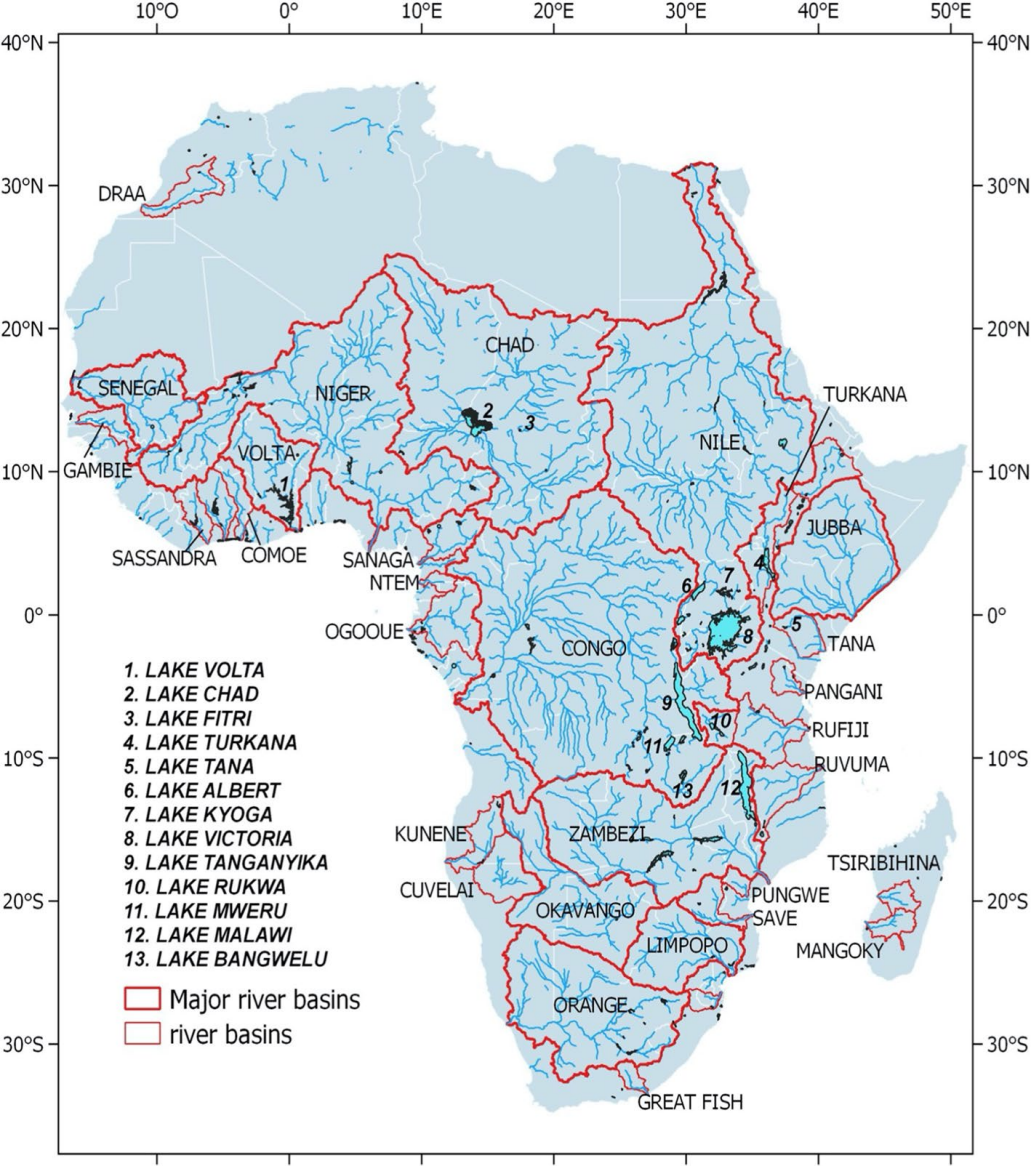
Location of river basins and lakes in Africa

The African continent hosts some of the largest freshwater systems worldwide (Fig. 1), including the Nile, the longest river in the world, and the Congo River, the second-largest world’s basin both in terms of drainage area and discharge to the ocean (Dai et al. 2009; Laraque et al. 2020). Three of the 10 largest freshwater lakes on Earth, in terms of area and volume, are also located in Africa, namely the Victoria, Tanganyika and Malawi Lakes (Hernegger et al 2021). Further, Africa is home to many wetlands and floodplains in the Congo, Chad, Niger, Okavango and Niles basins, which are of global significance for biodiversity and the carbon and nutrient cycles (Simaika et al. 2021; Lunt et al. 2019; Hastie et al. 2021). Some regions are also largely covered by tropical forest that harbors incredible natural resources and acts as a carbon sink that stores billions of tons of carbon (Verhegghen et al. 2012; Dargie et al. 2017). Conversely, smaller water systems, such as streams, reservoirs, ponds and tanks, are also part of the African landscapes (Gardelle et al. 2010), providing water, food and natural resources for agriculture to a large portion of the population that remains mainly rural such as in the sub-Saharan region.

The hydrology of the African continent is characterized by a wide range of processes that are under the influence of complex atmosphere–land–ocean interactions and the availability of freshwater is strongly heterogeneous, generally highly seasonal and driven primarily by local or remote rainfall (Conway 2002; Conway et al. 2009). It is subject to strong climate variability across timescales (Janicot, 1992; Hulme et al. 2001), from interannual to decadal changes (Ropelewski and Halpert 1996; Stager et al. 2007), with alternate periods of floods or droughts (Tierney et al. 2015; Oguntunde et al. 2018). Among many examples, Lake Chad and its dramatic shrinkage since the 1980s is obviously a symbol of how climate variability can impact African freshwater resources (Pham-Duc et al. 2020).

In the past decades, the combined effect of climate change and anthropogenic pressure made freshwater availability a current global major concern (Alcamo et al. 2007; Hoekstra et al. 2012; Famiglietti 2014; Konapala et al. 2020). In rapidly growing African economies, increasing demands for freshwater supply to sustain population growth and the needs of the agriculture and industrial sectors (Haddeland et al. 2014; Mehran et al. 2017) now pose significant threats to water resources. Environmental alterations such as land use practices, groundwater stress and deforestation, along with political conflicts, transboundary rivers, inadequate infrastructure and low adaptive capacity in many regions, make the African population particularly vulnerable to hydro-climatic variability and to any future changes in the water cycle (Inogwabini 2020; Anderson et al. 2021).

A better understanding of hydrological processes across the continent therefore becomes fundamental for addressing these current challenges, for reducing uncertainty in future evolutions of water availability and for developing mitigation strategies (Adenuga et al. 2021).

Surprisingly, the hydrology of the African river basins remains one of the least studied worldwide and has not attracted as much attention among the scientific and international communities (Alsdorf et al. 2016) as has been, for instance, for other large tropical and subtropical regions such as the Amazon River basin (Fassoni-Andrade et al. 2021) or the Indian sub-continent with the Ganges–Brahmaputra (Papa et al. 2015 and references therein). It currently leaves an insufficient knowledge of Africa hydro-climate characteristics.

Historically, the monitoring of land surface water variability relies on in situ observations that quantify the movement (height, extent, discharge) and quality of water in river channels, lakes and wetlands. However, in situ networks are sparse, unevenly distributed globally, or even within a hydrological basin, particularly in remote areas with difficult access or security concerns. In situ gauge networks are generally costly to maintain, especially for developing countries, and the availability of ground-based hydrological information has dramatically decreased during the last decades (Fekete et al. 2012), especially over Africa (Tramblay et al. 2021). A striking example is the Congo River basin, where hydrological monitoring can be traced back to the beginning of the 20th century (Trigg and Tshimanga, 2020). Until the end of the 1960s, more than 400 gauging sites provided observations of water level and discharge (Alsdorf et al. 2016), while today, there are only 15 gauges considered as operational (Laraque et al. 2020). In addition, in many places, even when data exist, their public access can be restricted by government agencies (Chawla et al. 2020) and they are often not available to the scientific community due to political situations or transboundary water sharing conditions (Papa et al. 2010a). Finally, in situ data are not capable of monitoring all water characteristics such as large floods events, wetland-river connectivity or the variability of numerous small lakes/ponds in a same area (Alsdorf et al. 2007).

In addition to the assessment of water stock and fluxes, water quality is also a major issue. For example, cyanobacteria, often referred to as blue-green algae, are opportunistic prokaryotes with very strong adaptive capacities, some species of which can synthesize highly toxic metabolites. Cyanobacterial bloom can lead to eutrophication of water bodies. They can disrupt the dynamics of aquatic ecosystems by reducing water clarity and leading to hypoxia and therefore death of fish and benthic invertebrates following the degradation of cyanobacterial blooms. The production of toxins may disrupt irrigated crops (Mhlanga et al. 2006), recreational uses and may constitute an acute health risk (Funari and Testai 2008) by inducing digestive or neurological diseases.

Monitoring suspended particulate matter (SPM) in inland waters is also fundamental. SPM is related to the sediment fluxes in rivers, lakes, and reservoirs and can help with assessing the sediment discharge, and more generally the sediment budget within catchments, including its seasonal variability and its evolution over time. In turn, the sediment budget is controlling the silting rate of the dams, which impacts the sustainability of hydroelectric structures and the supply of water for treatment plants. SPM in surface waters also contributes to pollution and public health issues, playing an important role in the transport of nutrients and various types of contaminants (World Health Organization 2018). Indeed, SPM commonly favors bacteria development and, at the same time, decreases their mortality through ultraviolet protection (Rochelle-Newall et al. 2015). Some of these bacteria or microbes cause widespread water diseases like diarrhea, which is one of the major causes of mortality in children under five years in developing countries (Reiner et al. 2018). Despite sub-Saharan Africa being the world region with the highest burden, few studies have analyzed the link between water quality parameters and bacteria in this area (Levy et al. 2016).

In this context, satellite remote sensing techniques offer a cost-effective means for monitoring the various components of the terrestrial water cycle, with a relatively continuous, high spatiotemporal coverage and reasonable accuracy (Chawla et al. 2020). Over the last thirty years, they have been very useful to hydrology investigations with the advent of monitoring the extent and elevation of water bodies and their changes over time from space (Alsdorf et al. 2007; Calmant et al. 2008; Chawla et al. 2020; Fassoni-Andrade et al. 2021, among others). For instance, since the late 1990s, radar altimetry missions provide observations of water levels of lakes, rivers and floodplains (Cretaux et al. 2017) under their orbit tracks, now with the potential of long-term monitoring at thousands of virtual stations (VS). In parallel, the use of satellite observations in a wide range of the electromagnetic spectrum (visible, infrared, and microwave, and their combination) has been developed to monitor the extent and quality of surface water bodies for various spatial and temporal scales (Papa et al. 2010b; Pekel et al. 2016; Prigent et al. 2016; Huang et al. 2018). They are often complementary to observations from the Gravity Recovery And Climate Experiment (GRACE) mission which provides, since 2002, long-term time series of the spatiotemporal variations in total terrestrial water storage (TWS) (Tapley et al. 2004) changes and helps to depict emerging changes in water availability at the global scale linked to environmental or human disturbances (Rodell et al. 2018).

In parallel, the availability of remote sensing observations has fostered the developments of hydrological and hydraulic modeling that helps to understand hydrological processes and their interactions and to build scenarios of past or future hydrological evolutions in the context of climate change, environmental alterations and flow/storage regulations by human interventions (Sood and Smakhtin 2015; Lettenmaier et al. 2015; Döll et al. 2016).

Earth Observation advances are therefore a game changer for surface hydrology and a unique opportunity to develop comprehensive observing systems to monitor Africa’s land water and manage its water resources.

**Fig. 2:**
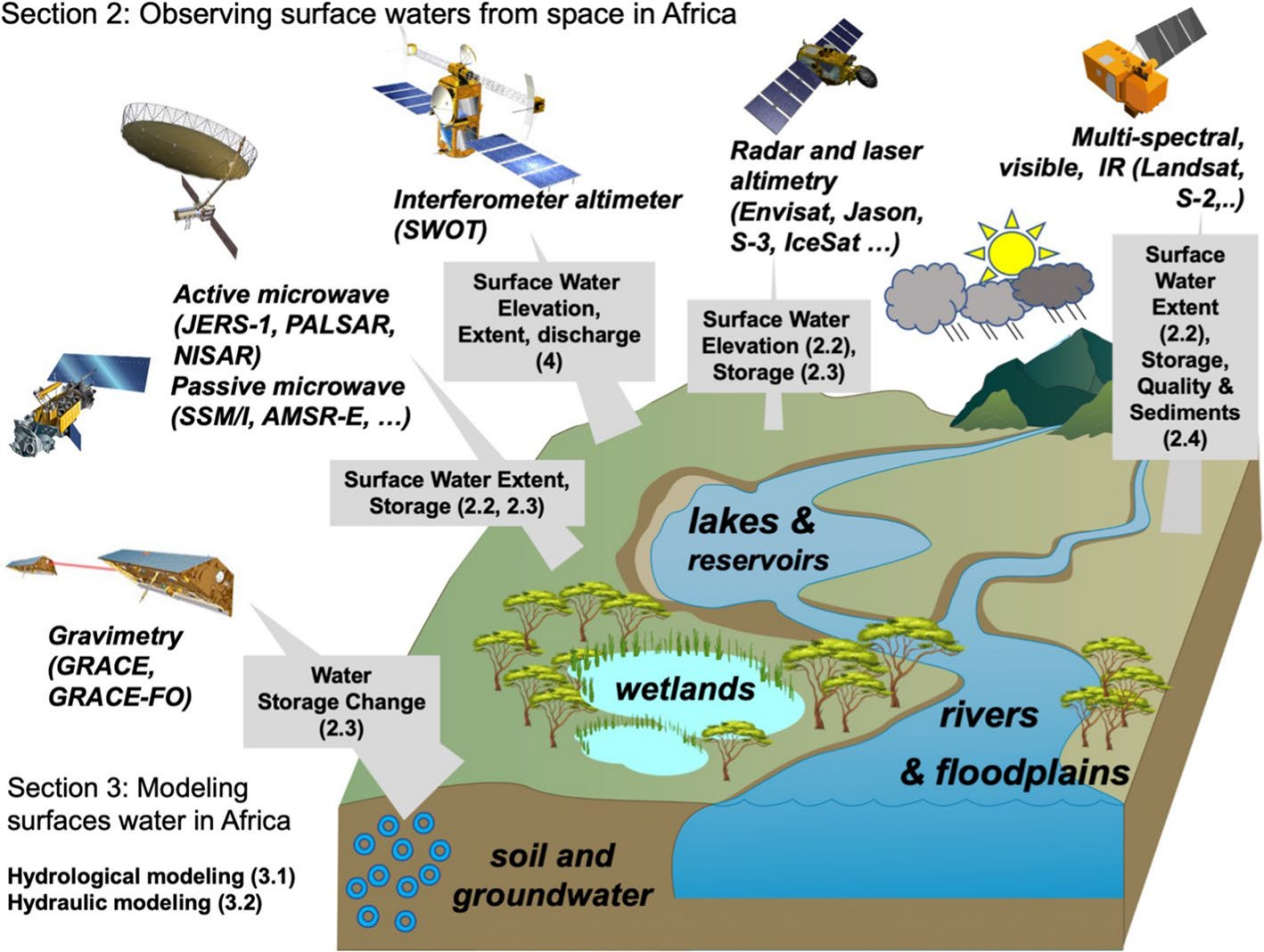
Schematic representation of the outline of the review. The hydrological variables (water elevation, extent, storage, quality) of the surface water components and the main sensors/satellites that are used to monitor them are indicated. The number in bracket provides the section which addresses the respective hydrological variables and remote sensing techniques

In the present paper, we review the developments and achievements of more than three decades of advances using remote sensing for surface waters in Africa (Fig. 2), highlighting the current benefits and difficulties.

Our review accounts for the various variables (water elevation, extent, storage, quality) of the surface water components including lakes, rivers, floodplains and wetlands. We analyzed approximately 200 publications. These contributions were selected using the knowledge of the experts that were reunited to make this review and using databases such as Web of Science. We mainly consider published papers in peer-reviewed journals and we do not include reports and other non-research articles and activities such as magazines, newsletters, editorials. Conference papers of notable relevance are also included. The period covered is from ~ 1990 to present. For each variable (Fig. 2), we summarized how it is retrieved from remote sensing observations, and present and discuss the advances that have been achieved from this information. Therefore, we show how the availability of a large number of sensors and observations, from low to very high resolution, coupled with models offers new possibilities to monitor a continent with scarce gauged stations. We also discuss how the use of satellite observations could benefit societal applications such as water resources management, flood risk prevention and environment monitoring. We then present perspectives, currently fostered by the upcoming launch of dedicated surface hydrology satellites, such as the Surface Water and Ocean Topography (SWOT) or the NASA-ISRO Synthetic Aperture Radar (SAR) mission (NISAR) or the planning of future missions for the next decade, such as the SMall Altimetry Satellite for Hydrology (SMASH) constellation and Sentinel-3 Topography New Generation (S-3 Topo NG).

The review is organized as follows (Fig. 2). Section 2 reviews the various methodologies and results in monitoring surface water from space. Section 3 deals with hydrologic and hydraulic models applied to African basins and with the use of remote sensors. Section 4 presents the current observational challenges and the future opportunities of the use of Earth Observations in hydrological sciences and multi-disciplinary sciences and for societal applications resources management. Finally, Sect. 5 concludes the study and provides recommendations.

## Observing Surface Waters from Space in Africa

### Surface Water Elevation from Earth Observation

Surface water elevation of inland water bodies is a key parameter in hydrology and water resources and has been long measured through historical gauge networks (Fekete et al. 2012). Over Africa, where traditional in situ measurements are sparse, a comprehensive monitoring of surface water elevation still remains a challenge. Moreover, the lack of fiducial in situ measurements can possibly be a limitation regarding the ground validation of remote sensing products.

Although, up to now, no satellite mission has been specifically designed to survey inland water elevations, satellite altimetry has been extensively used in the last three decades to remotely sense surface water elevation variations in lakes, rivers, reservoirs, and floodplains (Calmant et al. 2008) and improve the long-term monitoring of Africa’s hydrology.

Radar altimeters onboard satellites are initially designed to measure the ocean surface topography by providing along-track nadir measurements of water surface elevation (Stammer and Cazenave 2017). Over the continents, pioneering studies in the Amazon basin (Fassoni-Andrade et al. 2021) quickly demonstrated the capacity of retrieving accurate surface water elevation from radar echoes and adapted retracking procedures at the intersection of the satellite ground track with a water body (Birkett 1998; Frappart et al. 2006; Da Silva et al. 2010). These intersections, named virtual stations (VSs), are defined by the satellite orbit configuration, the repeat cycle of which define also the temporal interval sampling of altimetric observations (generally 10, 27 or 35 days) (Normandin et al. 2018; Papa et al. 2012a). At a given VS, surface water elevation is estimated through the inversion of the signal round-trip propagation time between the satellite and the Earth’s surface, which provides the range. Several corrections (in hydrology, essentially due to delayed propagation through the atmosphere or the interaction with the ionosphere and geophysical corrections due to the dynamics of Earth’s surface) need to be applied to this range to retrieve the surface water elevation. The height of the reflecting surface, given in respect to the height of the satellite above the reference ellipsoid, is then corrected from the local undulation of the geoid in order to be converted into an orthometric height or geoidal altitude of the water body, the variable that is useful for hydrologists. For a detailed and complete description of the characteristics of the different satellite altimetry missions and the estimation of surface water elevation for hydrology, including the various re-trackers used, altimetric waveform description, the specific corrections (geoid gradient for lakes, hooking effect for rivers and small water bodies), the associated errors and biases, the different acquisition modes [Low-Resolution Mode (LRM), SAR, SAR Interferometric (SARIn)], we refer to Cretaux et al. (2017).

Several groups or institutions around the world provided time series of surface water elevation, covering a wide range of water bodies and applications. Table 1 provides the main sources and databases (non-exhaustive) that provide surface water elevation time series over Africa.

**Table 1:** List of main databases providing surface water elevation time series over inland water bodies from radar altimetry

Name of the database (types of water body)	Producer (reference)	Website
River & Lake (Rivers, Lakes, reservoirs)	De Montfort University (Berry et al. 2005)	http://altimetry.esa.int/riverlake/continent/africa.html
Hydroweb (Rivers, lakes reservoirs)	IRD/LEGOS, CNES (French Space Agency), and Universidade do Estado de Amazonas (Cretaux et al. 2011; Da Silva et al. 2010)	http://hydroweb.theia-land.fr/
DAHITI (Rivers, lakes reservoirs)	German Geodetic Research Institute (Schwatke et al. 2015)	https://dahiti.dgfi.tum.de/en/
G-REALM (Lakes and reservoirs)	USDA NASA (Birkett et al. 2017)	https://ipad.fas.usda.gov/cropexplorer/global_reservoir/
GRRATS (Rivers)	Ohio State University (Coss et al. 2020)	http://research.bpcrc.osu.edu/grrats

The in-depth assessment and validation of the water levels derived from the satellite altimeter over lakes, rivers and other inland water bodies were performed worldwide against in situ gauges (Frappart et al. 2006; Da Silva et al. 2010; Papa et al. 2010a; Ričko et al. 2012; Cretaux et al. 2018; Kao et al. 2019; Paris et al. 2022; Kittel et al. 2021a; Kitambo et al. 2021), with satisfactory results and uncertainties ranging between few centimeters to tens of centimeters, depending on the environments (Cretaux et al. 2017).

*Radar altimetry over African lakes* The large lakes (Fig. 1) of Central Africa (Chad, Fitri) and East Africa (in the Rift and adjacent regions, such as Lakes Victoria, Tanganyika and Malawi) are icons of Africa’s tremendous water resources and valuable sentinels of the effects of climate variability and change. They remain highly dependent on the long-term rainfall characteristics (Becker et al. 2010; Hernegger et al. 2021), and they also influence the climate locally (Nicholson and Yin 2002). For instance, rainfall over Lake Victoria was found to be 30% higher than in the surrounding land areas, since part of the evaporation returns directly to the lake through precipitation (Asnani 1993; Anyah et al. 2006). Synchronous variations in water level records on the three largest lakes in the Rift region are explained by large-scale climate mechanisms associated with oscillations of warm and cold phases of El Niño (Ogallo 1988; Nicholson 1996; Ropelewski and Halpert 1996; Stager et al. 2007) or the Indian Ocean Dipole (IOD) (Hastenrath et al. 1993; Marchant et al. 2006), or the Atlantic SST (Sea Surface Temperature) anomaly patterns (Janicot 1992). The IOD is also partly driving the lake water level over East Africa, modulated by El Niño-Southern Oscillation (ENSO) events (Mistry and Conway 2003; Birkett et al. 1999). Tierney and Russell (2007) further highlighted the significant influence of the Indian Monsoon variability and the migration of the Inter Tropical Convergence Zone (ITCZ) on African lake levels. The use of an 80 year-record of in situ level data at Lake Malawi (1916–1995) helped with identifying the links with the rainfall variability over the catchment and the flows of the Zambezi River (Jury and Gwazantini 2002), consistent with large-scale climate variability that helps the prediction of lake levels a year in advance. Finally, other studies have shown that global warming has an impact on precipitation and temperature variations with consequent changes in African lake levels (Xu et al. 2005; De Wit and Stankiewicz 2006).

Despite the importance of a regular monitoring of lake levels in Central and East Africa, very few lakes of this region are instrumented with level gauges allowing long-term monitoring.

Radar altimetry helps to fill this gap and has contributed to a better understanding of the processes that drive water level variations in these lakes. For example, Mercier et al (2002) used seven years of radar altimetry data with TOPEX/Poseidon (T/P) over the Great Lakes and large-scale rainfall measurements to reveal the links between SST (Sea Surface Temperature) in the Indian Ocean and rainfall over East Africa, which generated the consequent variations in the levels of these lakes. Combining satellite altimetry with GRACE observations and rainfall data, Becker et al (2010) confirmed that the variation in total water content over East African watersheds and lakes (Victoria, Tanganyika, Malawi and Turkana) was closely linked to the IOD and ENSO, confirming early results from Birkett et al (1999).

Using radar altimetry observations among other data, Swenson and Wahr (2009) evidenced a larger impact of human activities (such agriculture practices and the construction of dams) on Lake Victoria, between 2002 and 2008, as compared to other natural lakes in the region. They also showed that only 50% of Lake Victoria’s water level variations could be explained by natural causes, while the other half is entirely due to discharges into Lake Kyoga directly downstream. In particular, radar altimetry data revealed that between 2002 and 2008, Lake Victoria’s water level decreased about 40% faster than that in Lakes Tanganyika or Malawi.

Ahmed and Wiese (2019) also used GRACE data in combination with time series of lake water level changes from the Hydroweb database (Cretaux et al. 2011) to determine how much of the Total Water Storage (TWS) variability in East Africa and in the Zambezi basin comes from the lakes, including Lake Malawi. For the period 2002–2018, they report an increase in the water volume of eight lakes, which contributes to ~ 60% of the overall TWS increased over the Rift zone. This change is largely driven by the water volume variations over Lake Victoria. On the contrary, the region of Lake Malawi experienced a decrease in TWS over the same period, but with a large interannual variability, alternating water mass loss and gain over the catchment. Lake Malawi variations explain 50% of these regional changes, mainly due to natural climate variability.

Recently, Hernegger et al. (2021) used in situ data and satellite altimetry from the Database for Hydrological Time Series of Inland Waters (DAHITI) database (Schwatke et al. 2015) to evidence very high-water levels on six Kenyan lakes in the central rift valley, linked with the very high positive rainfall anomalies during the 2010–2020 decade, especially after 2018. This study, which covered a very long period (1984–2020), concluded that the recent water levels have reached record highs, due to very high rainfalls over the region in the past years.

Over Central Africa, in the Lake Chad basin, the origins of the lake level variations are different and have been directly influenced over the last decades by the succession of drier and wetter periods, but tempered by a strong interaction between surface water and groundwater (Pham-Duc et al. 2020).

**Fig. 3:**
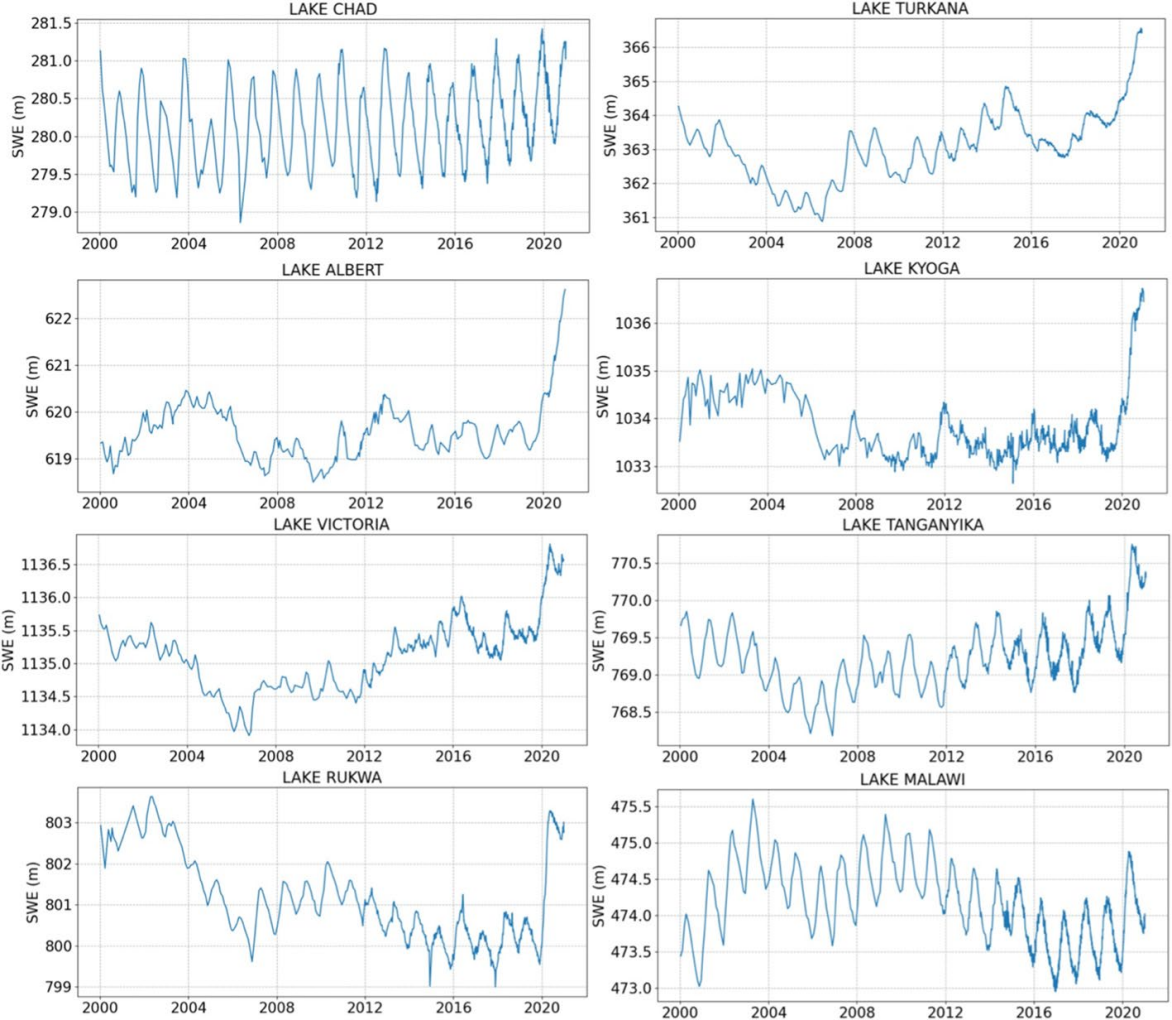
Surface water elevation for 2000–2020 for a selection of eight large lakes in Central, East and Southern Africa (See Fig. 1 for their locations), extracted from the Hydroweb database (hydroweb.theia.land.fr) indicates the square of Pearson's correlation coefficient

To illustrate the use of radar altimetry over the lakes, Fig. 3 shows water level variations for eight large lakes across Africa (see Fig. 1 for their locations) over 20 years (2001–2020) derived from observations available on Hydroweb (Table 1). For lakes of such large size, many studies have evaluated the accuracy of altimetry-derived water level variations using field data at several locations worldwide in different environments. It showed that altimetry makes it possible to obtain long time series with a temporal resolution of a few days, and an accuracy of a few centimeters (Ričko et al. 2012; Cretaux et al. 2018).

Figure 3 shows a strong seasonal signal on Lakes Chad and Malawi, whereas for the Rift lakes, the signal is primarily dominated by multi-annual variability. Three generic behaviors are observed. For the first one (Lake Chad), we observed a gradual increase in water level, more pronounced at the end of the period (2019–2020). The second behavior, for the lakes in the Rift Valley (Victoria, Tanganyika and Turkana), is characterized by a very sharp increase at the end of the period. Finally, Lakes Rukwa, Kyoga, and Albert show a strong positive anomaly during the years 2001 to 2005, and in 2019–2020.

This can be explained by increasing rainfall observed in all central and east Africa over the last two years, generating a dramatic jump in water levels. It confirms the results found for lakes in Kenya by Hernegger et al. (2021) linked to rainfall excess. For the biggest lake of the region, Lake Victoria, with a total area of ~ 68,000 km^2^, the increase in water storage for a level increase of ~ 1.5 m in one year is nearly 100 km^3^. Lake Malawi, located within the Zambezi basin, presents over the whole period two positive anomalies, in 2002–2005 and 2008–2010, followed by a constant shrinkage until 2016, then a quasi-constant water level, only varying at seasonal scale, with a significant but not drastic increase as for the Rift valley lakes. This was in part observed in Ahmed and Wiese (2019) and fully linked to spatial patterns of the rainfall over the period of observation.

Other recent studies showed the potentials for retrieving water levels over smaller lakes, especially in western Africa, such as Lake Volta (Ni et al. 2017) and lakes in Nigeria (Okeowo et al. 2017). As discussed in Cretaux et al (2016) and Biancamaria et al (2016), one of the major limitations of satellite altimetry is its partial spatial coverage due to the satellite intertrack distance that leaves large regions with no observations, especially in the tropics (at the equator the intertrack can vary from tens of kilometers to few hundreds of kilometers), and prevent monitor of small lakes in between the tracks. This has improved with the tandem interleaved orbit configuration of Sentinel-3A/B (since 2018) which allows the densification of the Earth’s coverage and therefore targets a much larger number of lakes and significantly improves the survey of narrow reservoirs. More recently, laser altimetry techniques, such as measurements by ICESat-2, proved useful to monitor water levels and water storage variability in reservoirs and lakes at the global scale and allow highlighting the important contribution of human management in Southern and Eastern Africa (Cooley et al. 2021).

Over the next few years, as discussed later in Sect. 4, major improvements to monitor surface water elevation are expected from SWOT which, thanks to his wide swath coverage will observe all lakes with an area bigger than 250 m × 250 m globally.

*Radar altimetry over African River basins* In parallel to applications over lakes, radar altimetry has been long used for monitoring the river systems of Africa. Many studies were conducted over African river basins to evaluate the capability of radar altimetry to retrieve the variations in water river elevation, and validate the estimates with the available in situ networks or dedicated field campaigns to overcome the lack of ground measurements. These studies contributed to the global effort to assess the potential of the technique and foster methodological developments. Michailovsky and Bauer-Gottwein (2014) used ENVIronment SATellite (ENVISAT)-derived water level variations along the Zambezi River and obtained a root-mean-square error (RMSE) of 32–72 cm with respect to in situ gauge data. Evaluations have also been reported over the Congo (Becker et al. 2014; Paris et al. 2022), the Nile (Eldardiry and Hossain 2019), the Niger (Schröder et al. 2019) and the Ogooué river basins (Bogning et al. 2018), with relative error and uncertainties in the same range as for other basins worldwide (Frappart et al. 2006; Da Silva et al. 2010; Papa et al. 2012b). Normandin et al (2018), focusing over the Niger River, is probably the most comprehensive evaluation of the performance of altimetry missions over Africa, assessing seven satellite missions (i.e., European Remote Sensing Satellite-2 (ERS-2), ENVISAT, Satellite with Argos and ALtika (SARAL), Jason-1, Jason-2, Jason-3 and Sentinel-3A), against in situ records at 19 gauging stations in the Inner Niger Delta (IND) from 1995 to 2017. It reported an overall very good agreement between altimetry-based and in situ water levels with RMSE generally lower than 0.4 m. Better performances are reported for the recently launched missions such as SARAL, Jason-3 and Sentinel-3A than for former missions, suggesting improved results thanks to the use of the Ka-band for SARAL and of the SAR mode for Sentinel-3A. Similar conclusions regarding the improved performances of radar altimetry over time in a multi-mission context are also reported in the Congo Basin (Kitambo et al. 2021) where comparisons with long-term in situ water stages (1995–2020) provide RMSE reducing from 75 cm with ERS-2 to 10 cm with Sentinel-3A. We refer to Cretaux et al (2017) for a description of the recent missions and the advantages of the SAR mode as compared to the previous low-resolution data acquisition modes.

**Fig. 4:**
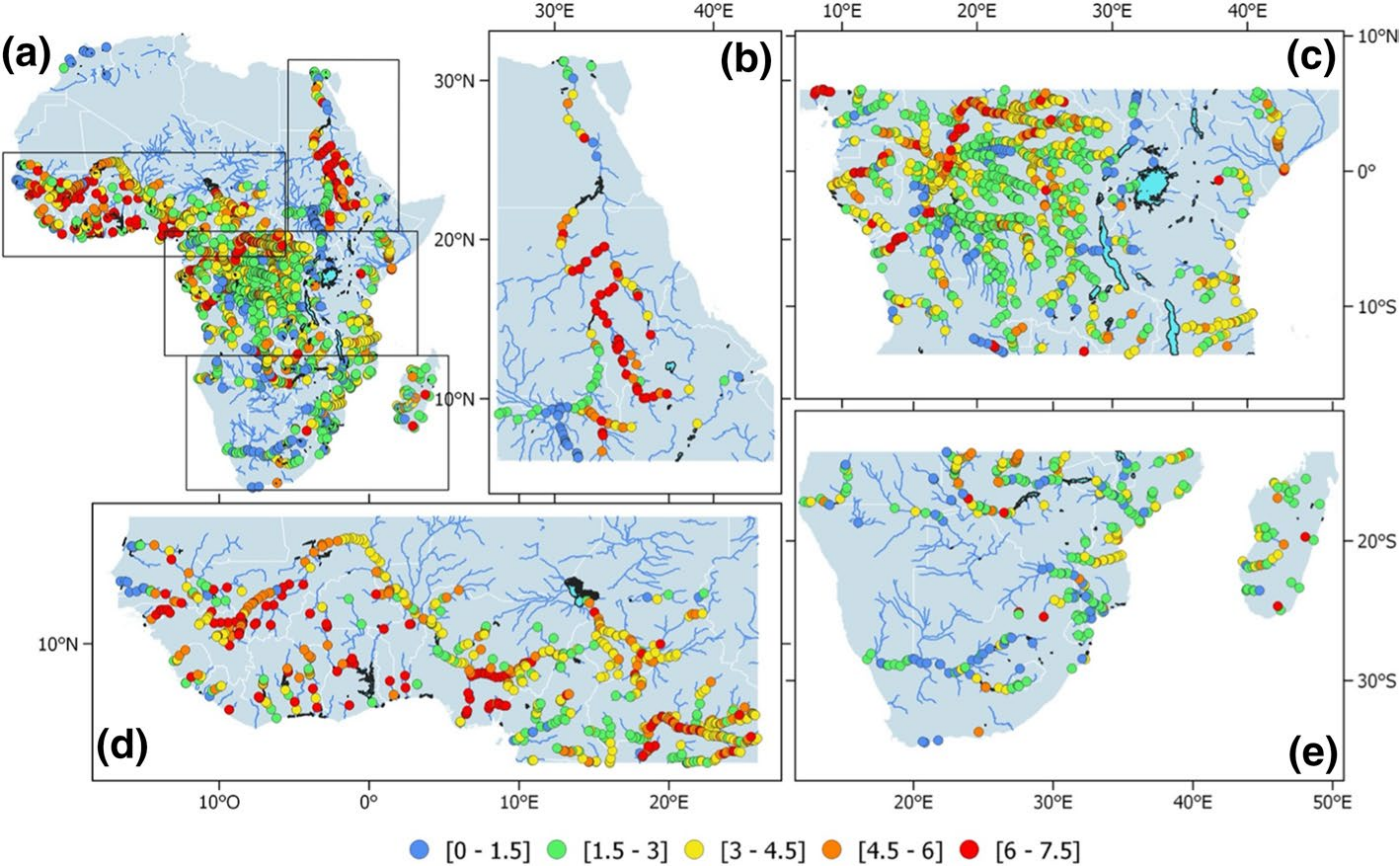
River maximum amplitude of surface water elevation at altimetry-derived virtual stations **a** over Africa (in meters) and zoom in over **b** the Nile region, **c** the Congo River Basin and Central Africa, **d** the Sahelian region, **e** Southern Africa and Madagascar

In recent years, many efforts have been seen carried out to increase the number of altimeric observations available across the African continent, fostered by the launch of new missions and as the result of almost thirty years of research and developments. But it is also a willingness of the scientific community, in agreement with local water agencies, to engage new studies specifically over Africa. Therefore, from several VSs a few years ago, there are now thousands of VSs available across the river basins of the continent, freely accessible on websites (Table 1), with potentially hundreds more of long-term observations. This is shown in Fig. 4 which displays the maximum amplitude of surface water elevation estimated for all available SV over Africa obtained from the Hydroweb database (see Table 1).

Note that all available VSs are displayed independently of the altimetry mission. So, it is worth noting that all SVs which are displayed don’t necessarily cover the same period of time and don’t have the same record length. Here, we used a total of 2157 VSs, including 173 VSs from ENVISAT (2002–2010), 204 VSs from Jason-2 (2008–2019), 230 VSs for Jason-3 (2016-present), 960 VSs for Sentinel-3/A (2016-present) and 797 VSs for Sentinel-3/B (2018-present). Moreover, for some VSs, the maximum amplitude is calculated from a time series with a 35-day interval sampling (ENVISAT), while others are estimated from observations with a 27-day (Sentinel-3A/B) or a 10-day (Jason-2/3) interval sampling. However, Fig. 4 clearly shows the high spatial coverage achieved over certain large river basins, but also on secondary size rivers.

**Fig. 5:**
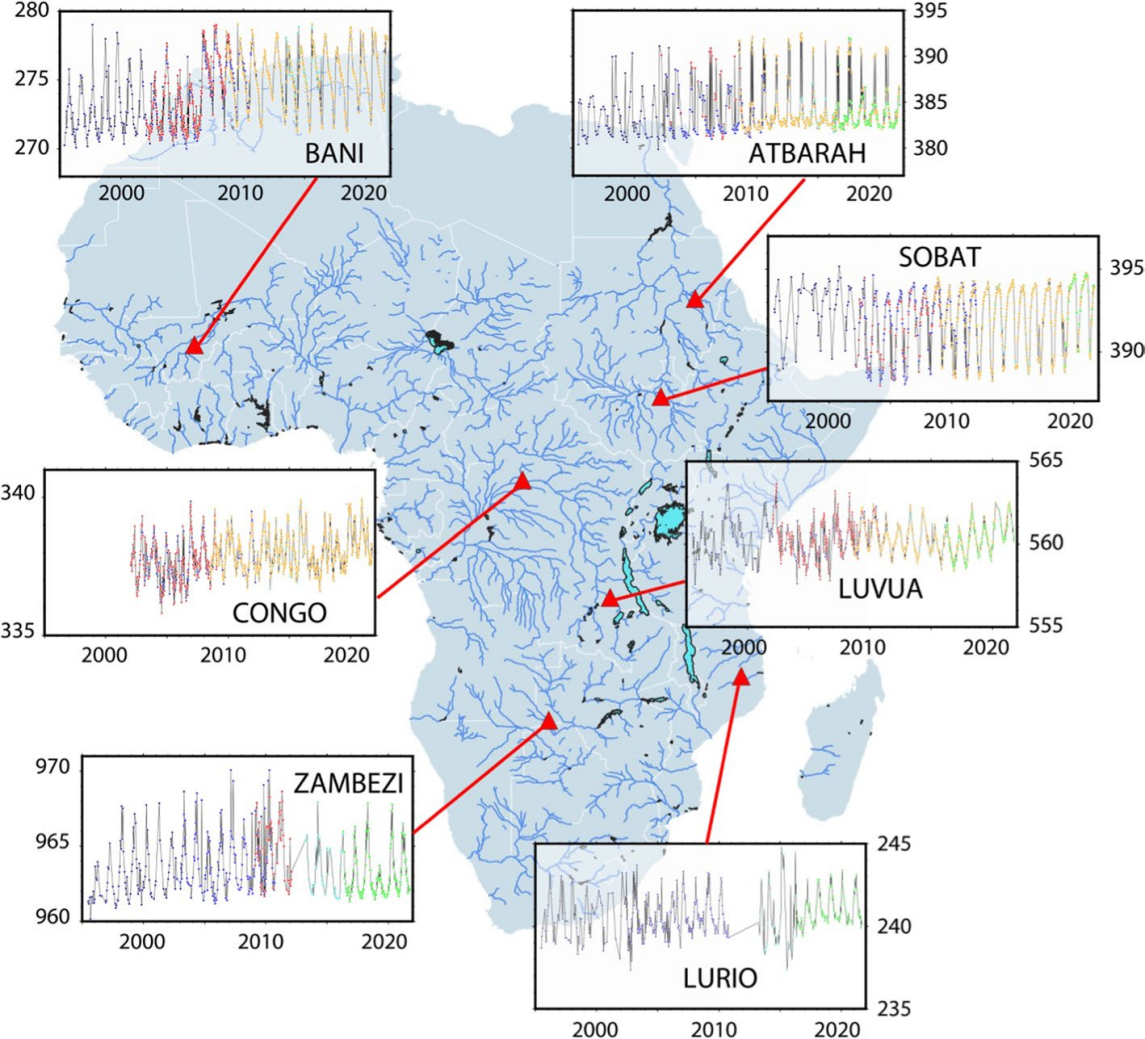
Examples of long-term time series of altimetry-derived river water elevation (in meters) at several locations in Africa from multi-mission observations. Dark blue stands for ERS-2 measurements (every 35 days from 1995 to 2002), blue for ENVISAT (every 35 days from 2002 to 2012), light blue for SARAL (every 35 days from 2013 to 2016), red for Jason-1 (every 10 days from 2001 to 2008), orange for Jason-2 and Jason-3 (every 10 days since 2008), green for Sentinel-3A (every 27 days since 2016) and Sentinel-3B (every 27 days since 2018)

To further illustrate the potential of radar altimetry measurements over the rivers of Africa, Fig. 5 shows examples of long-term time series of altimetry-derived river water elevation at several VSs in Africa obtained from the combination multi-mission observations.

The altimetry time series in Fig. 5 are composed from all the individual series collected by the successive altimetry missions since 1995, in a same reach. Bias between series due to river slope is taken into account to refer to a same location. Examples are provided for various environments and various types of rivers and geomorphological characteristics. For instance, the Atbarath River, a tributary of the Nile River, shows an example of river flowing in a desert environment and is characterized by reach width that varies largely between the low and high-water seasons (the minor bed is less than 100 m wide when the major bed is around 600 m). The Sobat river, also located in the Nile River Basin, is flowing in a vegetated area, with river width varying seasonally between ~ 100 and ~ 800 m. Over the Congo River, the time series illustrates the water elevation variability in the Cuvette Centrale, 1650 km upstream of the river mouth, characterized by a dense forest environment, frequently inundated. Luvua is a tributary of the Lwalaba River, in the Upper Congo, carrying waters from Lake Mweru to the Congo fluvial system. The reach is about 400 m wide, laterally bordered by seasonal inundated floodplains that can extent over several kilometers. As another example of large river, Fig. 5 also shows a reach of the Zambezi River located just downstream of the Sioma Falls, 2000 km upstream the river mouth, in a rare vegetation environment. The river It is well channelized with a width of ~ 1200–1300 m that stays almost constant over the seasons. Lurio is chosen as an example of small coastal river, flowing is a low vegetation environment, with a width seasonally varying between ~ 60 m and more than 800 m. Finally, the Bani River, a right-hand tributary of the Upper Niger river, upstream the Inner Delta is flowing in a quasi-desert environment. Its width can vary significantly, from 350 m at low waters to 1500 m at high waters when the major bed is temporarily inundated.

Altimetry data are extremely useful to better understand the processes that drive the hydrological variability across scales. Using T/P radar altimetry over the Chad Basin, Coe and Birkett (2004) demonstrated that river discharge upstream of the lake could be estimated from space, therefore, the lake and marsh height could be predicted in advance.

Becker et al. (2014) used radar altimetry-derived water levels at 140 VSs from ENVISAT to classify groups of hydrologically similar catchments in the CRB. In the Inner Niger Delta, cross-correlation analysis among water levels from the altimetry mission identifies time-lags between the upstream and the downstream part of the region of around two months that could be related to the residence time of water in the drainage area (Normandin et al. 2018).

In the Congo Basin, Lee et al. (2011) characterized the variability of terrestrial water dynamics in the Congo Basin using a combination of GRACE and satellite radar altimetry, while satellite altimetry-derived observations recently revealed the full range of variability of water surface elevation across the various rivers and wetlands of the Congo system (Carr et al. 2019). Now, more than 2000 VSs are available in the Congo Basin, sometimes covering almost 25 years of observations, thanks to multi-mission analysis (Kitambo et al. 2021). This has helped to reveal the seasonal dynamics of the Congo annual flood pulse, along with the relative contributions of the Congo sub-basins to the entire basin hydrology and the link with large-scale climate variability, including the annual bimodal pattern observed in the rainfall and discharge (Laraque et al. 2020). Smaller and ungauged basins are also strongly benefiting from the use of satellite altimetry observations such that the Ogooué River Basin in Central Africa (Bogning et al. 2018) or the so-called abandoned basins in terms of monitoring such as in Madagascar (Andriambeloson et al. 2020).

Finally, in addition to the most used missions for hydrology (such as Jason-2/3, ERS-2, ENVISAT, SARAL, Sentinel-3A/B), some studies are now making use of observations from Cryosat-2 and IceSat satellites to retrieve much detailed hydraulic characterization of the reaches such as over the middle Congo River (O’Loughlin et al. 2013) or the Zambezi River (Kittel et al. 2021a) or to take advantage of the drifting orbits (Bogning et al. 2018).

In addition to nadir altimetry, SAR Interferometry (InSAR) is another technique that can be used to estimate changes in water level by determining phase differences of more than two complex SAR images obtained at different angles and/or at different times (Alsdorf et al. 2000). As firstly demonstrated over the Amazon, the technique provides water level changes in inundated floodplain vegetation under vegetation owing to the double-bounce effect (see Mohammadimanesh et al. (2019) for a review of the InSAR technique and its applications). Using this technique, Jung et al (2010) revealed strikingly different flood behaviors between the Amazon and the Congo systems, with the Congo system having less connectivity between the main channel, floodplains and interfluvial areas than in the Amazon. The complementarity between InSAR and ENVISAT radar altimetry has been also used in the wetland region of the Cuvette Centrale in the Congo Basin to derive surface water level from images from the Phased Array L-band Synthetic Aperture Radar (PALSAR) onboard the Advanced Land Observing Satellite-1 (ALOS) acquired from 2007 to 2010 (Lee et al. 2015) and to study the hydraulics characteristics of the Congo floodplain (Yuan et al. 2017a).

The new availability of such a large amount of altimetric-derived data over the African rivers opens new perspectives of scientific advances in hydrology, such as that in the Amazon Basin over the last decades. Among many examples, we can mention the use of satellite altimetry to derive altimetric profiles of rivers throughout the African river basins and provision of spatiotemporal variations in the water surface slopes which are useful to study backwater effects and their impacts on flows. Altimetry can also be very useful to observe channel-floodplain connectivity and study the role of channelized flows and of overbank flows, which contributes to surface water storage that has a crucial role in floodplain ecological productivity. The perspective to develop VSs over wetlands and flooded areas, far from river main channels, which seems now possible with Sentinel-3 SAR mode, will open new perspectives.

Further use of radar altimetry observations in hydrologic and hydraulic models is discussed in Sect. 3, while the role of water level derived from space to derive river discharge over Africa is discussed by Tarpanelli et al. (2021).

### Surface Water Extent from Earth Observation

The extent of surface water bodies and their variation is of primary importance to the water, energy and biogeochemical cycles and the maintenance of biodiversity of the African continent. In many regions of Africa, water bodies cover a large portion land’s surface area, with strong seasonal and interannual variability that play a key role in the carbon cycle (Hastie et al. 2021; Hubau et al. 2020), biogeochemistry processes (Borges et al. 2015) and the occurrence of water-related disasters such as flood risk in populated areas.

Measuring the distribution and variability in the extent of surface waters across different spatial and temporal scales has always faced great difficulties, especially using the in situ networks. It is indeed very difficult to estimate on the ground the variations in surface water extent in large floodplains and wetlands, especially under vegetation or during extreme events such as inundation. Even in the era of remote sensing observations, it still remains a challenge.

A variety of remote sensing techniques employing a wide range of observations in the electromagnetic spectrum, including visible, infrared, or microwave observations (Alsdorf et al. 2007; Prigent et al. 2016; Huang et al. 2018), have been developed in the last decades to estimate the distribution of surface water from very fine spatial resolution (few meters) to coarser ones (~ 50 km) covering various temporal scales and periods. These remote sensing techniques are now widely used in hydrology and water resource research.

Depending on the sensors or the applications they are developed for, these observations include a large range of space/time resolutions, most of the time resulting from a trade-off between temporal and spatial coverages and sampling (Chawla et al. 2020). For instance, SARs show very large capabilities to measure surface water extent at high resolution (10–100 m) but often suffer from a low temporal revisit time, making them not suitable to monitor rapid hydrological processes. In one hand, optical and infrared sensors have good spatial/temporal resolution, sometimes with ~ 10 m resolution and a few days revisit, but their capabilities are limited in tropical and subtropical Africa regions because of dense clouds and vegetation. On the other hand, passive microwave sensors offer frequent temporal sampling, sometime twice daily, but the observations are generally at low spatial resolution (~ 10–50 km) thus limiting their use to the monitoring of large surface water areas. As already mentioned in the previous section regarding surface water elevation, many methodologies regarding the monitoring of surface water extent from space in tropical environments have been originally conducted in the Amazon Basin (Fassoni-Andrade et al. 2021); with the African river basins only recently benefiting from these advances.

Following the use of SARs to delineate surface water extent in the Amazon floodplains and tropical forest environments (Hess et al. 1995, 2003), efforts have been carried out to undertake similar studies in the Congo River Basin that hosts several seasonally or permanently flooded areas, such as the Bangwelu and Upemba swamps or the Cuvette Centrale in the Middle Congo. Seasonal flooding dynamics and vegetation types were thus derived from the Japanese Earth Resources Satellite-1 (JERS-1) (Rosenqvist and Birkett 2002) or the use of multi-temporal SAR coverage, such as the ScanSAR mode of ALOS/PALSAR SAR data (Lee et al. 2011). They conclude that the JERS-1 SAR mosaics may serve well to appraise the maximum extents of flooding in the Congo River basin, but perform poorly to assess the dynamics and ranges of the variations. Bwangoy et al (2010) and Betbeder et al (2014) employed a combination of SAR L-band and optical imagery to characterize the Cuvette Centrale land cover. They estimated the wetland extent to reach more than 360,000 km^2^, representing about 32% of the total area.

Nevertheless, these datasets generally represent large data volumes and suffer from low temporal frequency observations that limit these monitoring to a few samples of a few basins, preventing systematic, long‐term assessments of inundation dynamics. This context is changing and SAR observations with a high resolution in the order of 10 m are becoming a very powerful tool to be used for monitoring flood risk and for managing disasters (Alfieri et al. 2018; Lindersson et al. 2020; Matgen et al. 2020). The case studies over Africa are becoming more relevant such as over Uganda (Barasa and Wanyama 2020), Namibia (Long et al. 2014) or the Zambezi region (Refice et al. 2020), even if SAR satellite missions, such as the Copernicus Sentinel-1 SAR (launched in 2014, global revisit of 6–12 days), still need to be fully exploited over Africa.

Passive microwave sensors [e.g., Special Sensor Microwave/Imager (SSM/I), Advanced Microwave Scanning Radiometer (AMSR-E)] have long demonstrated their capability for observing surface water and flood extent given the sensitivity of brightness temperatures and emissivities to the presence of surface water (Sippel et al. 1998; Prigent et al. 2007) and the different dielectric properties among water, soil and vegetation. They are not limited by cloud cover, with a near-daily revisit time (D'Addabbo et al. 2018), but suffer from a relatively low radiation intensity in the microwave spectrum that causes the spatial resolution of the data to be generally low (10–50 km), often insufficient to observe small water bodies. Floods can be assessed using passive microwave brightness temperatures (Brakenridge et al. 2007) calibrated with a ratio of dry/wet areas and river discharges to delineate flood plain extent and its variability and provide a fraction of surface water within an area. It was specifically applied for flood detection in some regions in Africa, and forecasting has been investigated in the Zambezi watershed (De Groeve 2010) using AMSR-E imagery or in smaller scale watersheds with sparse observations in Malawi, East Africa (Mokkenstorm et al. 2021). Products making use of passive microwave-derived observations have been also specifically developed to monitor surface water extent over large African basins, such as the Surface Water Fraction (SWAF) product based on multi-angular and dual polarization passive L-band (1.4 GHz) microwave signal from Soil Moisture Ocean Salinity (SMOS) (Fatras et al. 2021). This product has been recently used to map the spatiotemporal variability of water bodies in the Congo River basin at ~ 50 km spatial resolution and weekly temporal resolution from 2010 to 2017, with the ability of the L-Band frequency to retrieve water under dense canopy (Parrens et al. 2017). They reported that the mean flooded area of the Congo extent was between 2 and 3% of the entire basin. The dataset was also helpful to characterize flood and drought events in the basin during the last 10 years.

**Fig. 6:**
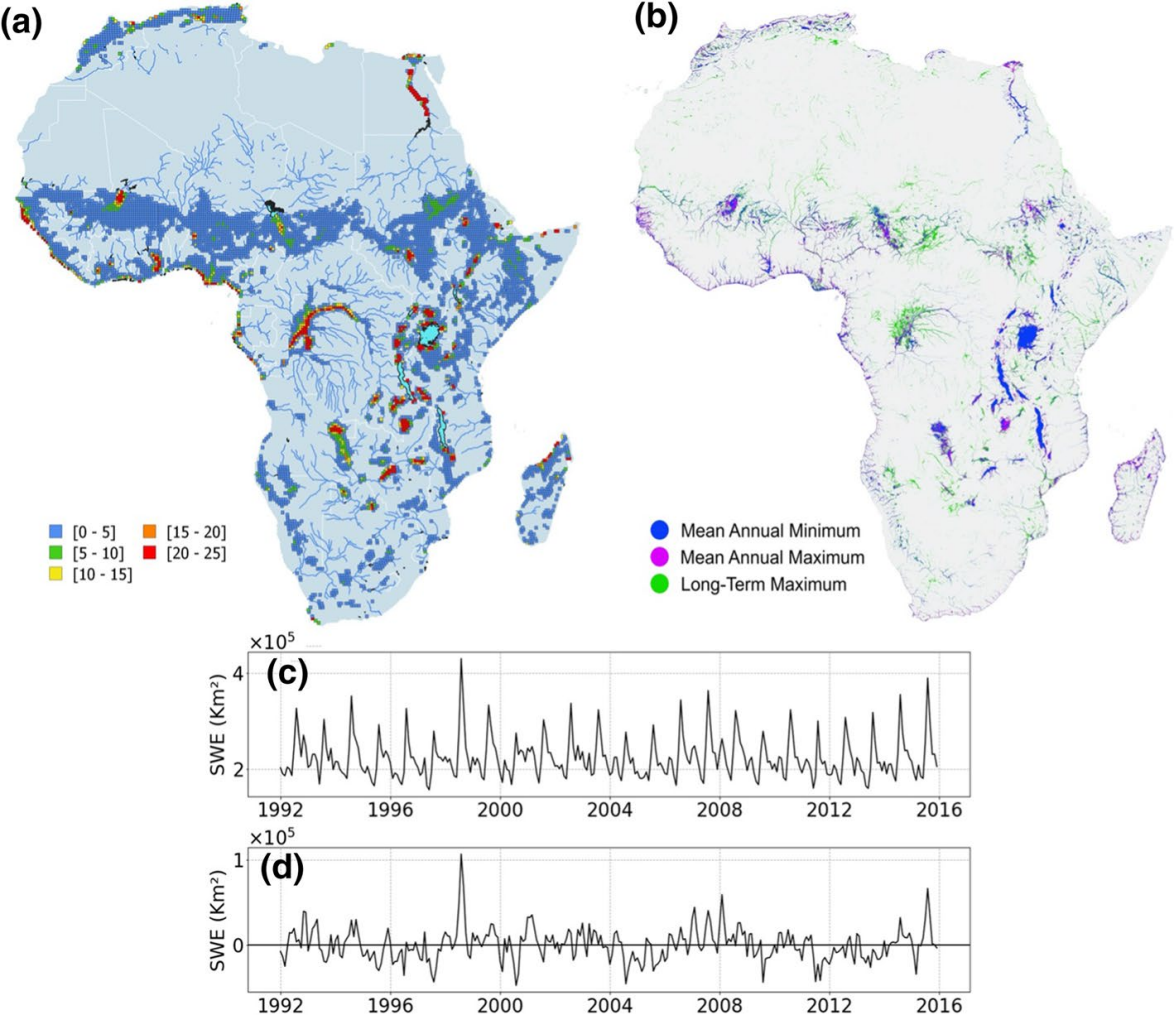
Characterization of surface water extent from multi-satellite techniques over Africa. **a** Mean surface water extent (1992–2015) from GIEMS-2 expressed in percentage of the pixel coverage size of 773 km^2^ (for visual purpose, only pixels with a value > 0.1% are displayed). **b** Surface water extent at 500 m spatial resolution from GIEMS-D15. **c** Time series of surface water extent aggregated over Africa, and **d** Corresponding deseasonalized anomalies obtained by subtracting the 24-year mean monthly value from individual months

The development of specific products for Africa remains limited and many studies over this continent rely on the use of products originally designed for global scale applications. These products are generally based on multi-satellite methodologies that combine the complementary strengths of the information provided by passive microwave observations with other types of satellite observations (Papa et al. 2010b). It includes the Surface WAter Microwave Product Series (SWAMPS) Inundated Area Fraction (Schroeder et al. 2015) and the Global Inundation Extent from Multi-Satellite (GIEMS; Prigent et al. 2007; Papa et al. 2010b) that quantify the variability of surface water extent at ~ 25 km over long periods (Papa et al. 2008; Prigent et al. 2012). The most recent version of GIEMS covers the period 1992 to 2015 on a monthly basis (GIEMS-2, Prigent et al. 2020), whereas SWAMPS now currently offers near-real-time information (Jensen et al. 2018). Figure 6 shows the distribution and temporal evolution of surface water extent estimated from GIEMS-2 (25 km, monthly) and GIEMS-D15 (500 m, static, see further below) over Africa, as well as their variability at seasonal and interannual timescales.

Over Africa, GIEMS was used to characterize the flood dynamics and seasonal flood wave in the Congo main steam and sub-basins (Becker et al. 2018; Kitambo et al. 2021). These studies also contributed to characterize basin scale water extent variability, including its link with large-scale climate modes (Becker et al. 2018), such as positive Indian Ocean Dipole events in conjunction with the El Niño event (especially the notable events in 1997–1998 and 2006–2007) that triggered major floods in the Congo Basin. It is worth noting that the estimates of surface water extent from SMOS, SWAMPS or GIEMS show an overall similar spatial distribution and variability in the Congo Basin. Their cross-evaluation against other products, such as the ESA‐CCI (European Space Agency-Climate Change Initiative), and IGBP water bodies products also shows a fair agreement among the estimates. Nevertheless, an assessment of these products against high-resolution SAR map over the region, as performed for example over the Amazon Basin (Prigent et al. 2007; Fassoni-Andrade et al. 2021), has not been done yet, leaving open the question of whether the maximum surface water extent from passive microwave products in the Congo is largely underestimating the actual water extent.

GIEMS was also used to monitor the extent variation in the Inner Niger Delta (Aires et al. 2014) and was the reference for water extent variability in climate studies over African wetlands (Taylor et al. 2018; Prigent et al. 2011). These studies analyzed the influence of wetlands on rainfall patterns across sub-Saharan Africa and demonstrated that rainfall in the vicinity of major wetlands was locally suppressed as compared to nearby drier areas, especially during the afternoon, highlighting the influence of sub-Saharan Africa wetlands on local rainfall patterns.

Estimations of surface water extent can also be retrieved using observations in the visible and infrared domain (such as from Landsat, Advanced Very High-Resolution Radiometer (AVHRR), Moderate Resolution Imaging Spectroradiometer (MODIS) or Sentinel-2A/B to name only the most relevant) which offer moderate to high spatial (~ 5–500 m) and temporal (daily to weekly) resolutions. However, in tropical and subtropical environments, these observations potentially show strong limitations for detecting surface water beneath clouds or dense vegetation. As a consequence, obtaining cloud-free data during the flood season of tropical Africa is a challenge (Klein et al. 2015; Huang et al. 2018; Mayr et al. 2021).

The potential for using the MODIS Terra instrument to monitor changes in flooding was demonstrated over specific regions such as the arid Inner Niger Delta (Bergé-Nguyen and Crétaux 2015). Weekly estimates make it possible to describe the process of inundation in the delta and to quantify the flooded scenes in terms of open water and a mixture of water, dry land and aquatic vegetation. More importantly, the study showed there was an increase in vegetation over the 14 years of study (2000–2013) and a slight open water decrease over the region. Pham-Duc et al. (2020) also used times series of the surface water extent derived from multispectral MODIS over the Lake Chad to show that the lake extent has remained stable during the last two decades (2000–2020) and has not been affected by drastic changes since the 2000s, despite some large year to year variations. However, this study also highlighted the need to provide a clearer definition of the observed target to accurately delineate water and water under vegetation, as differences between estimates across studies can remain large.

More recent satellite missions, such as Landsat 8 (since 2013), which carries the Operational Land Imager (OLI), Landsat 9 (since 2021, carrying OLI-2), Sentinel-2A (since 2015) and Sentinel-2B (since 2017), both carrying the MultiSpectral Instrument (MSI) (Drusch et al. 2012) offer new opportunities to study fine scale surface water extent variability. As proposed by Ogilvie et al (2020), the emergences of all these observations from Landsat 7/8, Sentinel-2 and MODIS offer new opportunities for multi-sensor approaches to long-term water monitoring of temporary water bodies, as demonstrated over the Senegal River floodplain. Their results provide important implications to guide the development of multi-sensor products to monitor large wetlands, floodplains and water bodies of Africa.

**Fig. 7:**
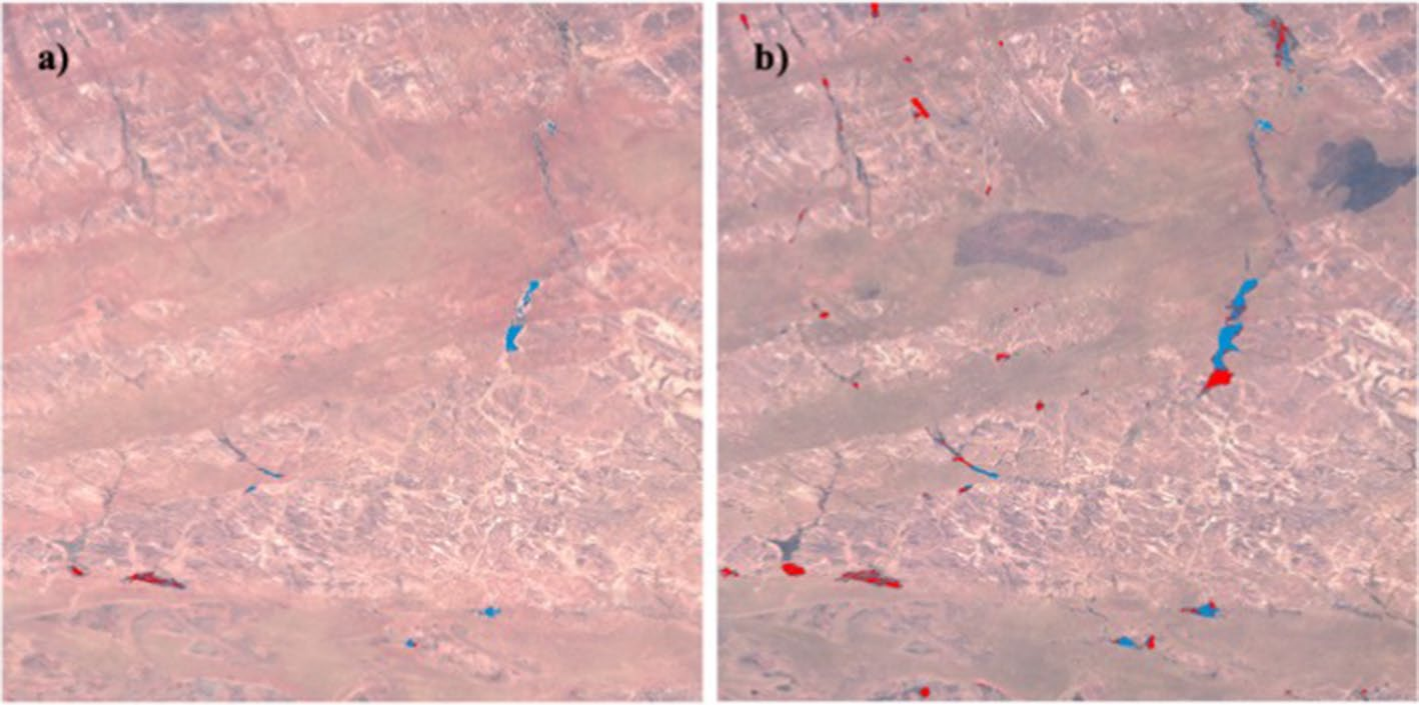
Sahelian small water bodies from Sentinel-2 and supervised classification in Gourma, Mali. **a** dry season (16/03/2018), and **b** rainy season (11/11/2018). Red: lakes with vegetation, Blue: open water

Finally, there is a strong need to accurately monitor the extent and variability of small water bodies, particularly in semiarid areas of Africa where these small water bodies, being widely spread all over the landscape, provide a critical resource to rural population. Small water bodies are very reactive to climate variability and can exhibit complex and sometimes unexpected dynamics, such the paradoxical increase in surface and volume during and after big Sahelian droughts (Gal et al. 2017). Optical sensors have proved successful to monitor their dynamics in different semiarid regions as for example the Sahel (Haas et al. 2009, 2011; Gardelle et al. 2010; Gal et al. 2016; Grippa et al. 2017), Tunisia (Ogilvie et al. 2018) and Namibia (Naidoo et al. 2020). Using Landsat, Pekel et al (2016) provided a detailed dataset, the Global Surface Water (GSW) of open inland water worldwide at 30 m resolution, that include the characteristics of the seasonal cycles and trends over the last 30 years. Although open water can be easily detected using the middle infrared bands, the retrieval of water areas is more difficult when aquatic vegetation or trees are present. Indeed, most small water bodies are not permanent and several of them are covered by aquatic vegetation and/or trees which requires the development of specific algorithms to take them into account. For example, a supervised classification (here, the Active Learning for Cloud Detection (ALCD) from Baetens et al. 2019) using Sentinel-2 over the Gourma region in the Sahelian Mali (Fig. 7, based on the T30PXC tile in the Sentinel-2 US-Military Grid Reference System (MGRS) naming convention) illustrates well the high number of small temporary lakes. In the middle of the dry season (Fig. 6a), only 158 lakes are depicted, mainly open water (150 open water, 8 with vegetation) for a total water area of 29,9211 km^2^. At the end of the wet season (Fig. 7b), 337 lakes have been detected, about half of them being covered with vegetation (177 open water, 160 with vegetation) for a total water area of 96,1802 km^2^. Only half of the detected lakes in Fig. 7b are included in GSW (Pekel et al. 2016), mainly due to the fact that this database focuses on open water and misses most of the lakes covered by vegetation. Maximum water area over the 1984–2019 period in this database is equal to only 67,3585 km^2^.

To conclude this section, it is also worth mentioning the efforts developed to take advantage of the combination of the various observations we described above. For instance, the low resolution but long-term estimates of passive microwave can be combined to optical data, such as those from MODIS with higher spatial resolution, but limited in time, via downscaling methodologies that combine both estimates into long-term, high-resolution products, such as the inundation extent product proposed over the Inner Niger Delta (Aires et al. 2014). This was one of the starting points of several other studies using downscaling approaches based on topography and floodability index, that now provide global maps of surface water coverage at high spatial resolution, such as GIEMS-D15 (Fluet-Chouinard et al. 2015; ~ 500 m spatial resolution) and GIEMS-D3 (Aires et al. 2017, 90 m) that can be extremely useful for African water bodies.

It is obvious that more investigations are required to fully characterize the diversity and variability in the extent of African water bodies, especially in relation to the physical processes driving this dynamic. Great opportunities, as discussed further in Sect. 4, will emerge with the upcoming launch of the SWOT (Biancamaria et al. 2016; Grippa et al. 2019) and L-band SAR from NASA/ISRO SAR (NISAR) missions which will provide improved monitoring of African surface water across many scales.

### Surface Water Storage in Lakes, Rivers, Wetlands and Reservoirs

Surface water storage, which represents the amount of freshwater stored in surface water bodies, is also a very important quantity to be estimated for hydrology and water resources (Schumann et al. 2016; Papa and Frappart 2021). As surface water storage is, by definition, linked to the variations in surface water level (Sect. 2.1) and extent (Sect. 2.2), it is similarly challenging to quantify at adequate time/space sampling.

The Gravity Recovery And Climate Experiment (GRACE) observations (and its follow-on mission GRACE-FO) provide, since 2002, estimates of the spatiotemporal variations in total terrestrial water storage (Tapley et al. 2004; Rodell et al. 2018) with an accuracy of ~ 1.5 cm of equivalent water thickness when averaged over surfaces of a few hundred square-kilometers. This quantity results from the changes in groundwater storage, soil moisture storage and surface water storage (it also includes ice and snow changes, but this is not applicable over Africa). Quantifying the storage of surface water is key to partition the GRACE-derived estimates in its different water storage contributions and to better quantify groundwater storage changes (Frappart et al. 2019). GRACE data also proved successful in estimating water stock changes over the Sahel (Grippa et al. 2011). In tropical and subtropical humid environments, the contribution of surface water storage to total terrestrial water storage can be substantial, sometimes up to 50% (Kim et al. 2009; Getirana et al. 2017a), and the lack of information at the surface prevents an estimate of groundwater/subsurface storage changes from GRACE data.

The estimation of surface water storage from space is generally based on multi-satellite approaches that use the complementarity between products which provide the dynamic of the surface water extent and observations of surface water elevation. Thus, their combination offers the possibility to estimate surface water volume changes (Cretaux et al. 2016; Papa and Frappart 2021).

Over lakes and reservoirs, satellite altimetry and satellite imagery together are now commonly used to calculate water storage changes in order to study their dynamic and water balance. For a detailed description of the concept that establishes the relationship between water level, extent and water volume changes in lakes and reservoirs using a hypsometry approach, we refer to Cretaux et al. (2016), Gao et al. (2012), Duan and Bastiaanssen (2013) and Gal et al. (2016). If the bathymetry of the lake or reservoir is known, this can also be used in combination with water level estimation to infer surface water storage variations.

Several databases, generally the ones that also provide water level from altimetry (Sect. 2.1), now make water storage change estimates in lakes and reservoirs available (Hydroweb and DAHITI for instance), including several water bodies in Africa. Notably, Tortini et al (2020) and Tusker et al. (2019) built on relationships between elevation and surface area from multiple satellite altimetry missions and surface water extent estimated from Terra/Aqua MODIS or Landsat (such as GSW). They estimate continuous surface water storage changes in large lakes and reservoirs globally for 1992–2019, with many targeted lakes in Africa.

Over rivers, given the heterogeneity of channels, streams and terrain, estimating surface water storage variations is very difficult and there are only several studies worldwide and over Africa (Papa and Frappart 2021) that have been performed so far. The estimation is based on the combination of inundation extent products with surface water elevation from altimetry, similar to lakes and reservoirs, but the difference in elevation within the basin or between rivers and floodplains needs to be taken into account. Over Africa, the studies were mainly focused over the Congo River Basin, considering surface water extent maps from high to low spatial resolution products, depending on the applications.

**Fig. 8:**
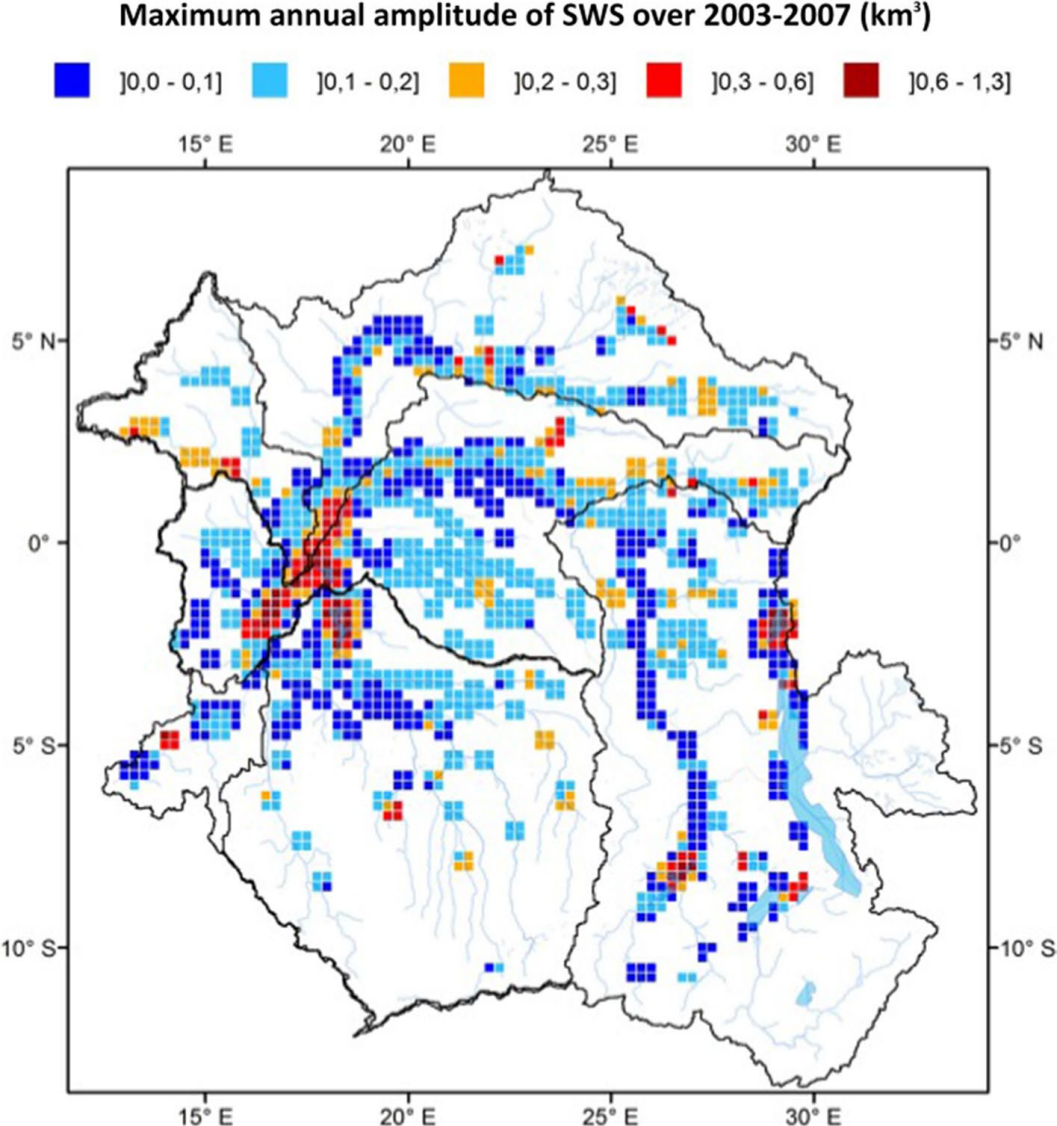
Maximum annual surface water storage amplitudes (in km^3^) over the Congo Basin. The estimates are obtained from a combination of surface water extent maps from GIEMS and water elevation from ENVISAT radar altimeter, over 2003–2007 (from Becker et al. 2018)

Becker et al. (2018) combined GIEMS observations with altimetry-derived water levels from ENVISAT at 350 VSs to estimate monthly surface water storage change over the period 2003–2007 (Fig. 8) following the development of the methodology over other river basins (Frappart et al. 2008, 2012; Papa et al. 2015). In general, the uncertainties associated with the method (calculated by taking into account the uncertainties on both the water level from altimetry and GIEMS surface water extent) is ~ 25% in tropical river basins (Frappart et al. 2008; Papa et al. 2015; Papa and Frappart 2021). Over the entire Congo Basin, Becker et al. (2018) reported that the mean annual variation in surface water storage change amounts to ~ 81 ± 24 km^3^ (with an estimated uncertainty of 26%). This variation accounts for 19 ± 5% of the annual variations in GRACE-derived total terrestrial water storage.

Lee et al. (2011) used a combination of several observations (including total water storage, water level from radar altimetry, precipitation and imagery from JERS-1 and MODIS) in a frame of a water balance study to estimate the amount of freshwater entering and exiting Congo wetlands. They showed that 111 km^3^ of water were flowing through the Congo wetlands each year. Over the Cuvette Centrale of the Congo (an area covering ~ 7800 km^2^), PALSAR observations in InSAR acquisitions were used in combination to ENVISAT altimetry to establish relationships between water depth and surface water storage and derived absolute surface water storage change over 2002–2011, with uncertainties of water storages ranging from 8 to 14% (Yuan et al. 2017b). But the difference in water storage and their variations in the region still vary depending on the methodology. For instance, the estimates of Yuan et al. (2017b) can differ up to 50% (average of ~ 30%) with the estimates from Lee et al. (2015). Frappart et al. (2021) suggested that the densification of the VS network over the Cuvette Centrale by including water elevation variations over the floodplains could impact the surface water storage by a factor four as compared to if only VSs over the rivers are used.

Over the Lake Chad region, Pham-Duc et al. (2020) combined MODIS-derived surface water extent of the lake and floodplains with water level from altimetry to estimate the variations in surface water storage in the area to be ~ 1.2 km^3^ annually (with a maximum error of 8.5% on this estimate). In combination with GRACE data, the study further showed that groundwater (estimated with a maximum uncertainty of 23%), which contributes ~ 70% of Lake Chad’s annual water storage change, is increasing since the 2000s, mainly because of sustained water supply provided by the two main tributaries of the lake. As a result, over the last two decades, Lake Chad is not shrinking thanks to groundwater and the tropical origin of water supply and is recovering seasonally its surface water extent and volume.

As we can see, most of these studies have been performed for large spatial scales and with temporal scales of monthly to seasonal changes and is restricted in general the data to capture only wide rivers, lakes and basin systems. Moreover, the spatial resolution of current gravimetry missions (generally hundreds of kilometers) is not adequate to infer changes in freshwater quantities at scales meaningful for to societal applications and water resources management. Nevertheless, these missions remain a powerful tool to study hydroclimatology and large-scale freshwater variability.

The forthcoming mission SWOT is expected to change the way surface water storage is observed globally, as it will provide, for the first time, direct estimates with an unprecedented spatial resolution (~ 100 m), even if the set of observables in floodplains and wetlands is still uncertain. A preliminary work carried out in the Gourma region lake (Grippa et al. 2019) has shown the potential of SWOT to follow the seasonal dynamics of water volumes in ponds and lakes in this area. However, deriving water masks in the Sahel may be difficult given that backscattering coefficients from water and land are sometimes close. In this case, coupling water levels by SWOT to water extent estimated by optical sensors could be a good option.

### Surface Water Quality from Water Color Measurements from Remote Sensing

While the above variables are more related to water quantity, Earth Observation can also be a powerful tool to monitor the quality of inland water, including sediments (suspended particulate matter-SPM), chlorophyll *a* (chl-a), cyanobacteria and colored dissolved organic matter (CDOM). In this section, we deal with water color techniques to monitor these parameters. Previous studies provided a comprehensive review of past, present, and new satellite sensors available for deriving water quality information in inland waters (Dörnhöfer and Oppelt 2016; Dube et al. 2015; Martinez et al. 2009; Pahlevan et al. 2017). Water color measurements are particularly challenging in Africa given on the one hand the atmospheric conditions (water vapor, desert dust, and biomass fires) which disturb the optical reflectance and on the other hand the strong seasonal dynamics of surface waters and the extremely high values of certain parameters, such as for example SPM (Robert et al. 2017).

*CDOM *CDOM is calculated based on absorption for a specific wavelength, often 440 nm. It is an indicator of the carbon content of surface water, so it plays a major role in understanding the role of surface water in the carbon cycle, particularly in the context of climate change (Kutser 2012; Williamson et al. 2009). However, in Africa, there are few studies on the satellite monitoring of this parameter.

*Chlorophyll a* Chl-a is an indicator of phytoplankton, and therefore of the primary productivity of lakes and it is one of the most monitored parameters using water color techniques in Africa (Shi et al. 2019; Dube et al. 2015). It provides information on the trophic state of the water and the risks of eutrophication. Mapping chlorophyll *a* (chl-a) is crucial for water quality management. Chl-a has absorption bands between 440 and 560 as well as at 670 nm (red band) and a strong reflectance at 500 and 700 nm. In inland waters, it seems preferable to use the red and Near InfraRed (NIR) bands and not the blue to limit interference with CDOM and SPM (Shi et al. 2019; Obaid et al. 2021). In Africa, chl-a studies have developed especially since the 2010s, particularly in the Great Lakes (Bergamino et al. 2010; Horion et al. 2010; Loiselle et al. 2014) and in southern Africa (Chavula et al. 2009; Chawira et al. 2013; Dlamini et al. 2016; Masocha et al. 2018) but more specifically in South Africa (Matthews et al. 2010; Matthews 2014; Malahlela et al. 2018; Sakuno et al. 2018; Obaid et al. 2021; Bande et al. 2018) and its use has since spread to other African countries. More recently, Buma and Lee (2020) showed the interest of using a 3-band algorithm to monitor chl-a in the Chad Lake with Sentinel-2 (Red bands and NIR band are used) and Landsat 8 (blue, green and red band) satellite data. Obaid et al (2021) on the Vaal Dam used a blue-green ratio for Landsat and red-NIR ratio for Sentinel-2 to retrieve chl-a. Gidudu et al (2021) on the Lake Victoria used a 488 nm/645 nm ratio with MODIS data. Normalized Difference Vegetation Index (NDVI) was also employed to monitor water hyacinth (Kiage and Obuoyo 2011; Dube et al. 2014; Shekede et al. 2008).

*Cyanobacteria* The use of the quantification of chl-a as an indicator of the abundance of cyanobacteria is problematic because this pigment exists for phytoplankton communities. Thus, PhycoCyanin (PC) is preferred, which is the unique pigment of cyanobacteria with an absorption at 620–630 nm. Finally, vegetation indexes can also be used [NDVI, Floating Algae Index (FAI), etc.] Despite the growing interest in estimating cyanobacteria in Africa (Dalu and Wasserman 2018; Ndlela et al. 2016), few studies focus on their monitoring by satellite (Stumpf et al. 2016), except in South Africa (Matthews 2014; Oberholste and Botha 2010). Matthews (2014) used the Maximum Peak Heigh (MPH) algorithm (Matthews et al. 2012) link to chl-a using Medium Resolution Imaging Spectrometer (MERIS) data to detect cyanobacterial blooms. The FAI (Hu 2009) is the most widely used spectral index for detecting dense cyanobacterial blooms (Shi et al. 2019).

*Sediments *The use of satellite data to monitor SPM was first applied to temperate coastal areas, and then ocean water followed by tropical areas. In Africa, its application to inland surface waters is very recent. Kaba et al (2014) and Robert et al (2016) used MODIS data to document SPM in the Tana (Ethiopia) and Bagré (Burkina Faso, north of Lake Volta in the Volta basin, see Fig. 1) lakes, respectively. Robert et al. (2017) assessed the capability of high (Landsat 7/8) and medium (MODIS) resolution satellite sensors to monitor SPM in extremely turbid waters in the Gourma region (Mali). Sentinel-2 data were also found to provide good results for SPM monitoring in Burkina Faso (Robert et al. 2021), over the middle Niger River and over the Lake Chad.

**Fig. 9:**
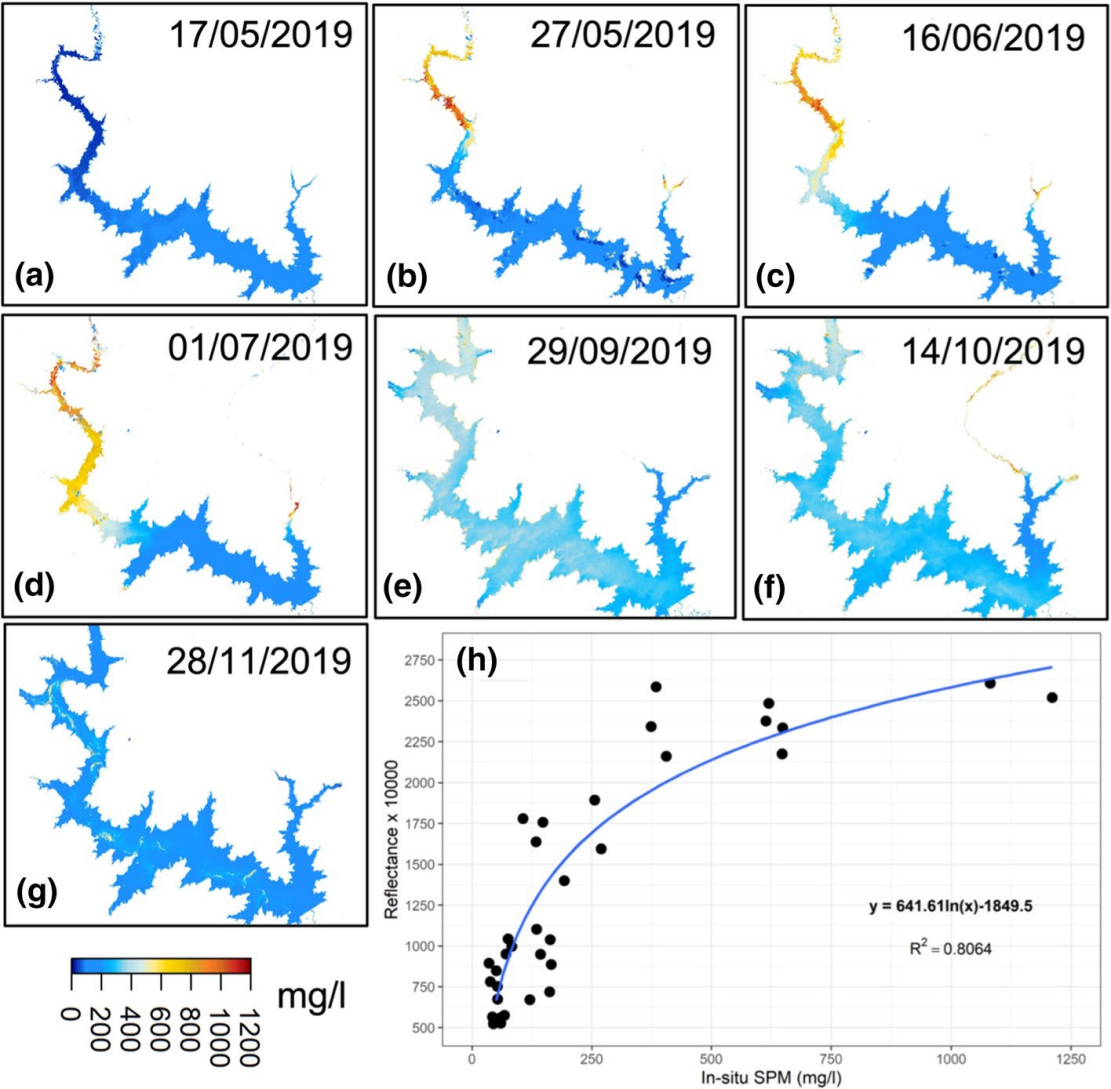
**a–g** Spatiotemporal evolution of suspended particulate matter (SPM) (mg/l) in the Bagré Lake (Near InfraRed (NIR) band inversed from Sentinel-2 images; **h** Sentinel-2 NIR band as a function of SPM in log *x*-axis scale. *R*^2^ indicates the Pearson’s correlation

All these studies are based on in situ measurements in order to identify the best corresponding band or ratio band to monitor the dynamics of SPM: Depending on the site, this is either the NIR/R ratio or most often the NIR band which seems the best to retrieve SPM values, in particular for high values. However, there is a limit at about 2500 mg/l above which reflectance reaches saturation. An example of the capability of Sentinel-2 to monitor SPM spatiotemporal variability in the Bagré Lake in Burkina Faso is shown in Fig. 9.

The NIR band derived from Sentinel-2 was used to study the SPM dynamics of the Bagré Lake (Fig. 9a–g). Before the start of the rainy season, low SPM values are observed throughout the lake. Then, the onset of the rainy season is synonymous with an increase in values in the upstream part of the lake. Then, it is like a wave of SPM which moves from upstream to downstream areas between the months of May and September. The usable images of 2019 make it possible to see this dynamic from the end of May to the beginning of July: with the highest values around 1000–1200 mg/l in the upstream part, 600–800 mg/l in the middle part, and lower values in the downstream part. The images of the end of September and the middle of October show a homogenization of the amount of suspended matter (400 mg/l) present in the lake. The rest of the year, the values are lower (less than 200 mg/l). It was also found that surface reflectance produced by the French Data Center THEIA provided better relationships to in situ SPM than surface reflectance produced by Copernicus which shows the importance of atmospheric corrections for water color-based applications (Fig. 9h). Finally, Robert et al (2021) highlighted that *E. coli* and diarrheal disease are strongly correlated with in situ SPM, and reflectance in the NIR which makes water color satellite approaches suited to monitor health hazard.

## The Use of Earth Observations for Modeling Surface Waters and Hydrological Processes in Africa

### Hydrological Modeling

Hydrological models are employed to numerically reproduce the hydrological functional behaviors and the water cycle processes through sets of mathematical equations. This representation of the processes related to the transition of rainfall to runoff through channels occurring at the land surface is termed hydrological modeling*,* which can also be understood as a means of quantitative prediction for decision-making (Tshimanga 2022). Hydrological modeling is commonly used to understand hydrological process, to predict hydrological variables, particularly when and where they are not currently measured in situ, and/or to predict the effects of future climate and explore “what-if” scenarios to understand the impacts of other changes, such as land use, dam building or urbanization. Quantifying all these processes is of paramount importance for hydrological prediction and water resources management under both stationary and non-stationary conditions, further complicated by our limited ability to measure or assess the subsurface interactions where many complex water fluxes take place.

In the African continent, a number of hydrological model applications have been initiated to meet the purpose of processes understanding and predictions across different scales, as well as water resources management plans. Some of these initiatives include the development of new models that are fit for purpose in the context of the African hydrology. Particular reference is made to the Pitman model (Pitman 1973; Hughes et al. 2007), the Agricultural Catchment Research Unit (ACRU) model (Schulze and George 1987) and a Hybrid Atmospheric and Terrestrial Water Balance (HATWAB) model (Alemaw and Chaoka 2003) that have been developed and used for simulating hydrological processes under different African hydro-climatic conditions.

A large number of hydrological modeling structures are currently available, but they differ in the degree of detail of the description of processes, the manner in which processes are conceptualized, the requirements for input and output data, and the possible spatial and temporal resolution. The common practice now in hydrological modeling communities is to use or adapt model structures that have been relatively successfully performed elsewhere. In this regard, a large number of global or land surface models have been used under different conditions and for different purposes across the African continent (Trambauer et al. 2013; Hughes et al. 2015; Boone et al. 2009; Grippa et al. 2017; Getirana et al. 2017b). Here, we do not intend to provide a classification of hydrological models, so more detailed information about the classification of these models can be referred to in the references cited.

Overall, several approaches have been used for hydrological models’ applications across the African continent. These approaches vary from parameter estimation (Nyabeze 2005; Kapangaziwiri and Hughes 2008), regionalization (e.g., Love et al. 2011), assimilation of new data types into hydrological models (Mekonnen et al. 2009; Milzow et al. 2011) and model uncertainty prediction (e.g., Katambara and Ndiritu 2009; Kapangaziwiri et al. 2012). Models have also been applied at different spatial scales from the relatively small to medium catchments (Hamlat et al. 2013; Gal et al. 2017; Nonki et al. 2021) to large rivers (Tshimanga and Hughes 2014; Casse et al. 2016; Munzimi et al. 2019) and even continental scales (Alemaw and Chaoka 2003; Trambauer et al. 2013). Apart from the general catchment water balance modeling, progress has also been made in the use of models for more specific processes such as flooding (Yawson et al. 2005; Ngongondo et al. 2013; Smithers et al. 2013) and drought assessments (Nyabeze 2004), modeling of floodplains (Birkhead et al. 2007; Unami et al. 2009), sediment yields (Ndomba et al. 2008) groundwater surface water interactions (Ayenew et al. 2008; Tanner and Hughes 2015). Models have also been used to address the purpose of water allocation (McCartney and Arranz 2007; Mwebaze et al. 2021).

In a review of the 16 most widely used hydrological models for water resources applications worldwide, with the purpose of assessing their suitability for drought forecasting in Africa, Trambauer et al. (2013) noticed that not all of these models sufficiently represent all the important water balance components for African semiarid areas. This is partly due to the fact that most models do not represent relevant hydrological processes that could be significant in these areas, such as transmission losses along the river channel, re-infiltration and subsequent evaporation of surface runoff, and interception of the wet surface, as well as the high temporal and spatial variability of rainfall. Out of the 16 well known hydrological and land surface models that were reviewed, they concluded that only five (i.e., PCRaster Global Water Balance (PCR-GLOBWB), Global Water Availability Assessment (GWAVA), land surface Hydrology Tiled European Centre for Medium-Range Weather Forecasts (ECMWF) Scheme for Surface Exchanges over Land (HTESSEL), Distributed Water Balance and Flood Simulation Model (LISFLOOD) and the Soil and Water Assessment Tool (SWAT)) were suitable for hydrological drought forecasting in Africa.

Results from the African Monsoon Multidisciplinar Analysis (AMMA) Land Surface Intercomparison Project carried out in West Africa have shown that general Land Surface Models (LSMs) have difficulties in representing surface runoff over the different West African soils and slow reservoirs (Boone et al. 2009; Grippa et al. 2017; Getirana et al. 2017b).

With a rapidly growing population and low level of water utilization, Africa’s economic development continues to be hampered by water policies and management decisions that are based on sparse and unreliable information (Guzinski et al. 2014) and a severe lack of in situ network already highlighted. Hydrological models have a particularly important role to play in water management in Africa in filling some of these data gaps. However, the lack of in situ data also severely hampers the development of reliable hydrological models, mainly due to the lack of calibration and validation data. This has been a continued challenge for hydrologists, even with advances in methods developed through the Prediction in Ungauged BasinS (PUBS) efforts (Hrachowitz et al. 2013).

Earth Observations have demonstrated their value globally in improving hydrological modeling (Lettenmaier et al. 2015), but in Africa, they play an even more crucial role. Almost all hydrological modeling undertaken on the continent now relies on Earth Observation for many of its data needs (Paris et al. 2022). While the growth in utilizing Earth Observation in hydrological studies only really took off in earnest since 2000, mainly due to the advent of satellite-derived data products (see Sect. 2), it has now become so ubiquitous to use Earth Observation in these studies that many hydrology papers no longer mention Earth Observation in the title (Lettenmaier et al. 2015).

Here, we provide some examples to illustrate the role Earth Observation currently plays in hydrological modeling at different scales and for differing applications over Africa. We use this to highlight some of the successes of applying these data in hydrological modeling of the continent.

One of the significant benefits of using satellite observations in hydrological modeling is the large spatial scale of what can be measured and used, although often at increased computational modeling cost. However, there are very few examples of hydrology modeling specifically for the African continental scale. This may in part be due to regional technical capacity issues, as well as data availability for such studies, but there has also been a tendency for researchers to develop global hydrology models, rather than just at a continental scale. Nevertheless, Earth Observation has contributed to studies specific to the African continent scale. One example of continent scale modeling is the use of daily remotely sensed surface water extent assimilated in the LISFLOOD hydrology model at the continental spatial scale in Africa and South America, with the conclusion that remotely sensed surface water extent improves streamflow simulations, potentially leading to a better forecast of the peak flow in ungauged regions (Revilla-Romero et al. 2016). These models have also been used to explore explicit links between hydrology and malaria transmission across the African continent (Smith et al. 2020).

Unsurprisingly, as observed in Sect. 2, Earth Observation has had much more research focus on hydrology at a large basin scale in Africa including Niger, Nile, Congo, Chad basins. This is perhaps the ideal scale for the use of satellite data in hydrology modeling, where the large scale of the basins makes in situ observations of sufficient detail hard to gather, as well as there being a better performance of hydrology modeling due to the longer timescale responses of such large basins. These large basins are also important transboundary river systems that require multi-country management and Earth Observation has played an important nonpartisan role in a more transparent understanding of the hydrology and water availability of these rivers (Ekeu-wei and Blackburn 2018; Guzinski et al. 2014).

A particularly well documented basin in Africa is the Niger River Basin, for which there have been many hydrology studies that benefited from satellite data. This basin is very particular and heterogeneous in terms of hydrological processes, since it involves tropical areas (e.g., the Guinean highlands), complex river floodplain systems (e.g., the Inner Niger Delta and the Niger river delta) and semiarid regions (e.g., the Red Flood tributaries). Pedinotti et al. (2012) for instance used satellite-derived observations of surface water (altimetry and GIEMS, see Sect. 2) to develop an improved version of the Interaction Sol-Biosphère-Atmosphère-Total Runoff Integrating Pathways (ISBA-TRIP) continental hydrologic system for the Niger basin, including a flooding scheme. As well as direct observations of surface water (see Sect. 2), Earth Observation is also crucial for primary model build datasets such as land use observations and for model driver datasets such rainfall. These models have used Earth Observation in the Niger Basin to explore a range of issues, such as the use of MODIS and Landsat for vegetation and surface water in groundwater recharge processes (Leblanc et al. 2007), and satellite rainfall products such as ECMWF Re-Analysis (ERA)-Interim for runoff estimation (Oyerinde et al. 2017) and the Climate Prediction Center morphing method (CMORPH), the near-real-time legacy product of Tropical Rainfall Measuring Mission Multi-satellite Precipitation Analysis (TMPA 3B42RT) and the Precipitation Estimation from Remotely Sensed Information using Artificial Neural Networks (PERSIANN) for flood forecasting (Casse et al. 2015). Casse et al. (2016) have employed the ISBA-TRIP model to investigate the long-term changes along the Red Flood tributaries, which are located in the Sahel region and have been facing intensified flood hazard over the last decades. A recent application for the Upper Niger River basin of the MGB model (MGB is the acronym in Portuguese for “Large Scale Hydrological Model”, Fleischmann et al. 2018) presented a two-way coupling of large-scale hydrological and hydraulic models over the basin and highlighted the importance of representing the interactions between floodplains and unsaturated soils during the annual flood wave propagation across the Inner Niger Delta.

The Nile Basin is another well-studied large basin in Africa, with many studies focusing on water resource management and rift valley lakes management issues. Digital elevation models (DEM) derived from radar observations (e.g., the Shuttle Radar Topography Mission, SRTM) have been used for basin delineation and flow routing to study lake hydrology in the upper Nile Basin (Bastawesy et al. 2013). Thermal infrared remote sensing data from AVHRR are used to estimate evaporation fluxes from the large Sudd wetland (Mohamed et al. 2006). Surface water extent observations from Landsat have been used to improve hydrology modeling of the lower Nile (Gleason et al. 2018). Recently, altimetry-derived surface water storage was assimilated into the World-Wide Water Resources Assessment (W3RA) model using the ensemble Kalman filter (EnKF) for the period of 2003 to 2016 (Khaki and Awange 2020), which improved model outputs, especially the surface water discharge RMSE which is reduced by approximately 33%.

Smaller African basins are also benefiting from modeling with a significant reliance on satellite dataset due to data scarcity, for example for modeling of flood hydrology and the subsequent flood hazard in the Oti River Basin (Komi et al. 2017), for water availability modeling in the Rokel-Seli catchment in Sierra Leone (Masafu et al. 2016) or for developing a water balance model for Lake Turkuna in East Africa (Velpuri et al. 2012), calibrated with a composite series of water level from T/P, Jason-1 and ENVISAT satellite altimetry data.

### Hydraulic Modeling

Although hydrological modeling includes the transport of water through rivers, either implicitly or explicitly, there is still a need for more specialist models that simulate in detail the flow transport in channels and floodplains. These hydraulic models, also commonly referred to as hydrodynamic models, generally utilize some derivative of the Saint–Venant shallow water flow equations and are used to model river channel and floodplain processes more explicitly and in higher detail than would typically be undertaken with a hydrological model. Importantly, they provide spatiotemporal predictions of water levels and velocities, which allows the mapping of floodplain inundation dynamics and simulate sediment transport characteristics. Satellite-derived observations of these parameters have also advanced our understanding of these processes and allowed calibration and validation where in situ data are sparce (Schumann et al. 2009).

Over the last decade, there have been an increasing number of Earth Observation-based hydraulic modeling studies carried out on large African rivers that can be more easily observed from space. These models have been used to investigate inundation dynamics, map flood hazard, and to advance flood modeling methods in data sparse contexts. Generally, the models utilize a global DEM to represent terrain and map floodplain and main river channels and rely on remotely sensed observations of water surface elevation and/or extent for model calibration and validation. Typically, these models also involve estimation of river channel bathymetry, often by treating it as a parameter to be calibrated alongside the friction coefficient. While the representation of bathymetry is crucial to the hydraulic model performance, observed bathymetry data are seldom available, which presents a key challenge of modeling large rivers in this data sparse context. The river flow data used to drive these models are acquired using a variety of methods, which are covered in the previous hydrological modeling section.

The Niger River again is a prime example of an African river whose study has been significantly enhanced by satellite data, having been the subject of no less than four hydrodynamic modeling studies within the last decade. The upper Niger, including the Inner Niger Delta, has been simulated with multiple models all of which make extensive use of satellite-derived water surface elevation and extent data, as described in Sect. 2 (Neal et al. 2012; Fleischmann et al. 2018; Haque et al. 2019; Getirana et al. 2021). Landsat imagery of water extents was used to estimate river channel width and validate modeled flood extents. Neal et al. (2012) also used water surface elevation observations from the Ice, Cloud, and land Elevation Satellite (ICESat) altimeter to calibrate their model’s friction and bathymetry parameters and showed the model was able to predict water surface elevation at altimeter VSs with a RMSE of 1.21 m. Fleischmann et al (2018) drew on water surface elevations from the Jason-2, ENVISAT and SARAL altimeters as part of their model validation process and found RMSE in modeled water surface elevation at altimeter VSs to be 0.95 m. The lower reaches of the Niger River including the Niger Delta have also been modeled hydraulically by Ekeu-wei and Blackburn (2020). They used ICESat altimetry to estimate bathymetry where observations were lacking and validated their model using flood extents from both MODIS and SAR observations. Their ‘percentage flood extent capture’ validation metric ranged from 70 to 92%.

Hydraulic modeling efforts on the Zambezi have also benefited from Earth Observation data. The Lower Zambezi flood forecasting model produced by Schumann et al (2013) is another example of a hydraulic model calibrated by varying channel friction and bathymetry parameters to fit water surface elevation observations from the ICESat altimeter. Model validation using flood extents obtained from Landsat imagery showed the predicted correct flood area to be 86%. Reaches of the Upper Zambezi and associated tributaries have also recently been modeled hydraulically by Kittel et al (2021b). In this instance, friction and bathymetry parameters were calibrated to observations of water surface elevation from CryoSat-2, and the model evaluated according to its ability to predict water surface elevation observed by the Sentinel-3 altimeter. The resulting RMSE values ranged from 0.6 to 1.31 m. Over the Congo River, the hydraulic modeling study by O’Loughlin et al. (2020) covers the Congo middle reach and major tributaries, where surface water elevation from the ERS-2 and ENVISAT altimeters were used to calibrate the friction and bathymetry parameters and validate the model (RMSE in modeled water surface elevation was 0.84 m). Landsat imagery was also used to obtain channel width estimates. Flood inundation fractions produced globally at 25 km spatial resolution from GIEMS (Prigent et al. 2007) were used to evaluate modeled flood extents, the dense rainforest here having prevented routine acquisition of higher resolution flood extents from satellite imagery. Also, Paris et al. (2022) presented the MGB hydrologic-hydraulic model for the whole Congo River basin, which was validated with several satellite-derived surface water elevation and extent datasets.

Published Earth Observation-based hydraulic modeling studies of rivers other than the principal African rivers described above are scarce but do exist. For example, Komi et al (2017) used the Oti River, a tributary of the Volta River in West Africa, as a case study for flood modeling of mid-size rivers in data sparse contexts. This river is typically around 60 m along the 140 km long modeled study reach, which is not wide enough to accommodate the footprint of a satellite altimeter. However, flood extents were observed by MODIS and were used to calibrate the model’s friction and bathymetry parameters. Fernández et al (2016) developed a LISFLOOD-FP (FP for FloodPlain) hydraulic model for the Logone floodplain in the Lake Chad basin, which was validated with 52 Landsat-based flood extent maps.

**Fig. 10:**
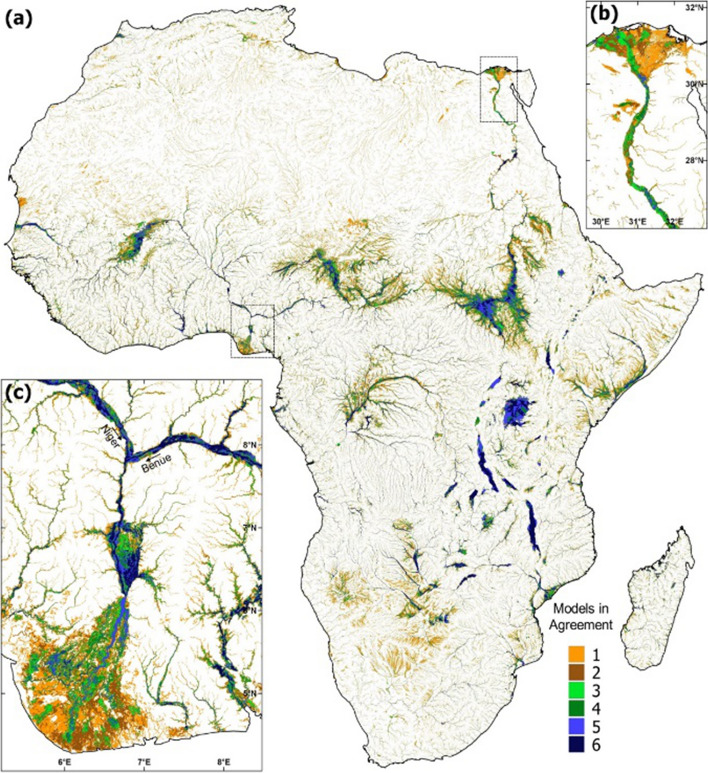
**a** agreement of Global Flood Models (GFMs) in Africa **b** Nile River, **c** Niger River (from Trigg et al. 2016)

There is a natural water cycle overlap between hydrology and hydraulic modeling and many studies that benefit from Earth Observation combine both approaches, for example for floodplain wetland management in Africa (Chomba et al. 2021). At the global scale, this can be seen through the development of Global Flood Models (GFMs) (Trigg et al. 2021), which would not be possible without increasing availability of satellite-derived open datasets. These GFMs have been a valuable source of currently missing flood risk information in Africa, nonetheless, these methods are still relatively new and there is some way to go before models consistently predict similar areas of flood hazard (Trigg et al. 2016; Bernhofen et al. 2018), as demonstrated in Fig. 10. In many locations, GFMs provide the best available information on flood hazard. However, these models are applied in an automated fashion and are generally not calibrated or validated, and their coarse scale can miss many smaller rivers (Bernhofen et al. 2021).

Despite the current limitations of GFMs, they can be of use to decision-makers, as documented by Emerton et al. (2020) in their evaluation of the use of GFMs in the emergency response to cyclones in Mozambique. This evaluation concluded that real-time hazard and risk information from a GFM provided a successful proof of concept with a positive real-world impact.

Specifically, the emergency flood bulletins derived directly from the GFM supported critical actions such as sending an assessment team to the region most likely to be affected. These extreme hydrological events are likely to become more common with climate change, and while Earth Observation cannot predict future climate change, it has been crucial for the development of models for predicting future impacts and will also be essential for monitoring the changing impacts over the coming decades (Guo et al. 2015).

There is no doubt that Earth Observation data have transformed hydrology and hydrodynamic modeling across Africa in the last few decades. With the development of new data sources, e.g., SWOT, NISAR, CubeSats, Unmanned Aerial Vehicles (UAVs) and smartphones, the future of modeling looks likely to continue this advance. This future will generate petabytes of data accessed through cloud storage and will require new methods of interpretation (McCabe et al. 2017).

## Observational Challenges and Future Opportunities to Monitor African Surface Waters: Way Forward to Water Resources Management

African surface hydrology and terrestrial water cycle have undoubtedly benefited from more than 30 years of developments and studies using Earth Observation and models, as demonstrated and discussed in the previous sections.

The use of remote sensing data has helped to improve our overall knowledge and understanding of the variability and changes of surface water across the African continent, as well as the processes and mechanisms that drive them at various scales.

Remote sensing data now represent a powerful complement to “classical” observations, and the synergy of both means is key to overcome difficulties in a region with sparse in situ networks and where many basins are still ungauged. This complementary exist in many aspects, since satellites often brings a mean to spatialize observations, over large-scale regions and sometimes at fine spatial resolution, while ground observations bring very accurate measurements with often high temporal frequency that no current satellite can compete with. Conversely, satellites give access to new quantities such as the inundated area, which are almost impossible to collect from the ground.

Therefore, remote sensing data dedicated to hydrology have strongly contributed to filling some gaps in the observational systems needed to accurately and comprehensively monitor the environment of Africa. Our review thus highlighted the main remote sensing products that are now available to study African surface water and how their use has contributed to new findings and advances in hydrology across the region.

*Current limitations* However, there are still many gaps that can be identified regarding our current knowledge of African surface water and the scientific community will need to tackle them in the near future to ensure a comprehensive monitoring and understanding of African hydrology. Some of these basic questions were listed in a community-based effort that synthetized 23 Unsolved Problems in Hydrology (Blöschl et al. 2019) which was recently further developed for Africa specifically through the “23 Unsolved Problems in Hydrology in Africa” initiative (https://www.researchgate.net/project/23-UPH-in-Africa).

Without the pretention of making an exhaustive list of these incomplete knowledges, one can note from our review analysis some obvious and important gaps regarding surface water. For instance, many water bodies are still left with no observations or are monitored with temporal/spatial sampling which are not adequate to capture their characteristics or dynamics. Our review clearly demonstrated that radar altimetry is a very powerful for monitoring water bodies such as lakes and reservoirs located under the satellite tracks, but it is still blind to many smaller lakes and reservoirs that stand in between the ground-tracks. Some processes and mechanisms that drive the variability of water bodies also require observations with high temporal frequency which most current satellite missions don’t offer. These limitations therefore prevent a better understanding of the variability of water level and extent in small lakes, reservoirs, rivers and wetlands and how these water bodies interact in complex systems such as in the river floodplain and in river–lake continuums. Additionally, there is still a very limited knowledge of water level and slopes at fine spatiotemporal resolution over African surface water, as well as topography in floodplains and flooded forests, preventing to significant improvements in hydraulic modeling of the systems. Similarly, river discharge, which was historically one of the first variables measured in hydrology and the most used to develop and calibrate models, is still not properly measured from space, as discussed in Tarpanelli et al. (2021). This review stresses a need to accurately estimate river discharge across the continent with fine spatial and temporal resolutions.

Our review also emphasized that, surprisingly, despite its importance, surface water storage in lakes, reservoirs, rivers and floodplains and its variations and changes still remain widely unknown in many parts of Africa. Such a conclusion regarding the surface freshwater reservoir is all the more striking given that, already more than fifteen years ago, Alsdorf et al. (2007) emphasized that “*Given societies’ basic need for freshwater, perhaps the most important hydrologic observations that can be made are of the temporal and spatial variations in water stored in rivers, lakes, reservoirs, floodplains, and wetlands.*” Lacking information on the amount of water stored and moving through surface water bodies prevents progresses in understanding their role in transporting sediment and organic matter, especially between channels and floodplains and how they impact the carbon balance, biogeochemical cycle and river ecology. The amount of water transported through floodplains, wetlands and lakes also impacts basin hydrology due to storage effects and regulates the size and timing of the river flood waves. Water storage is also key for water resources management and, as already mentioned, accurate partitioning of GRACE-derived total water storage into groundwater estimates requires measurements of surface water storage variations. Furthermore, it requires better spatial and temporal resolution than what is currently available in order to infer changes in freshwater storages at scales meaningful for to societal applications.

Finally, we identified a strong need for monitoring water quality through remote sensing, which remains challenging especially given the complexity of African inland waters. Using recent optical sensors, suspended sediment concentration could be retrieved with promising results over specific sites, but developing general algorithms to estimate this variable at the regional or continental scale remains a challenge. As part of river hydrology that involves flood dynamics, surface types and complex interactions, sedimentation processes need to be properly monitored to assess geomorphological changes in floodplain and river channels. Improving the observations of phytoplankton and the chlorophyll a dynamic using satellites is also identified as key since they contribute to healthy ecosystems, biodiversity and water security.

A better characterization of these components from space in the near future could foster new developments and improvements in modeling, especially regarding flood storage, parameterization of surface/subsurface processes and wetlands/rivers/lakes connectivity.

Satellite observations are also very important to quantify and understand how the hydrological cycle responds to environmental changes and alterations, due to climate change and anthropogenic pressure. For instance, human activities can alter the ecological services of rivers, lakes and floodplains by modifying their connectivity and the role of storage in modulating fluxes of waters, sediment and nutrients, with strong impacts on the environment. The construction of dams, the impacts of deforestation, and the alteration of flowing rivers (Grill et al. 2019) can also have strong implications on surface water and hydrology. Unlike the Amazon Basin (Fassoni-Andrade et al. 2021), where many studies have been conducted to investigate the interplay between environmental impacts and the water cycle, the African river basins still suffer from a lack of such assessments. Such investigations need to be developed and required long-term monitoring in addition to local assessment achieved on the ground. Satellite observations dedicated to hydrology could offer this unique opportunity for a continuous and long-term monitoring of environmental changes.

*Future opportunities* In the coming years, new opportunities will soon emerge thanks to an unprecedented and increasing monitoring of African surface water as upcoming and future satellite missions dedicated to surface hydrology will be launched.

The upcoming Surface Water and Ocean Topography (SWOT) satellite mission (Biancamaria et al. 2016) is now planned to be launched at the end of 2022, in collaboration between the United States National Aeronautics and Space Administration (NASA), the Centre National d’Études Spatiales (CNES, the French Spatial Agency), the Canadian Space Agency (CSA) and the United Kingdom Space Agency (UKSA). SWOT can be seen as a “topographic imager” satellite mission that offers observations at ~ 100 m spatial resolution thanks to its SAR interferometry technique and will provide measurements of surface water elevation, slope and water mask, with a temporal resolution of two measurements every cycle of 21 days, almost globally. More details about the mission and SAR interferometry and the KaRIn measurements on board SWOT can be found in (BIancamaria et al. 2016). Its capability to measure height and surface changes will help characterize variations in river discharge and lake water storage in all rivers wider than 100 m and water bodies greater than 250 m × 250 m in area under the swath coverage. A preliminary study on SWOT applicability over Sahelian lake has indeed shown promising results (Grippa et al. 2019). SWOT could also potentially provide measurements in wetlands and floodplains, even in regions with denser vegetation, although it is still unknown to what extent the observations will be affected. The mission will open new perspectives in a large range of applications, from scientific studies to water management since, for the first time, components of the water cycle such as global discharge of rivers, water storage changes in natural and artificial reservoirs, and the dynamic of floodplain storage will be observed jointly. In addition, SWOT data will offer new opportunities in the fields of data assimilation into models (Wongchuig-Correa et al. 2020) or across interfaces to study land–ocean exchanges (Stephens et al. 2020).

Because SWOT data will be freely accessible, as altimetry has always been, a major advantage of SWOT mission will also reside in the possibility to propose an independent, reliable, and accurate measurement system to scientists and water management authorities over an entire basin. This is of particular importance in Africa given the transboundary character of many of its river basins making Earth Observation a fundamental tool to fulfill the disparity in data availability among countries. Nevertheless, it should also be noticed that the SWOT mission will still face some limitations, especially regarding its use for water resource management or when it comes to the monitoring of surface water bodies such as wetlands, floodplains or inundated area with vegetation. SWOT will indeed provide water stage and discharge measurements on a global scale every 21 days (thanks to its wide swath technique, this revisit time will be reduced to 10–11 days in tropical regions) with a possible latency time of 3 days. This is a major improvement as compared to current observations but such temporal revisit might not be adequate for applications such as flood forecasting (Falck et al. 2021) or reservoir/dam operating. Moreover, in floodplains and wetlands environments, characterized by large extents of open water and sparse vegetation, SWOT will potentially provide observations of water surface elevation, extent and storage. However, in regions with denser vegetation, to what extent SWOT observations will be affected by vegetation is still largely unknown and more investigations are required. Therefore, studies that use the synergy with other satellite observations, which will be simultaneously available with SWOT, should be encouraged and supported to improve our understanding of the entire continental water extent and storage variability.

The capability of measuring surface water from space will also expand soon with the upcoming launch of the NASA-ISRO SAR mission (NISAR), a mission with an ability to map the extent of surface water in lakes and rivers, track floods, even under cloud cover and vegetation canopies and measure changes in the water surface height. SWOT and NISAR will complement the current capability offered by the COPERNICUS program to monitor surface water over long time periods.

As already stated, finer temporal sampling of the estimates of surface water elevation, ideally daily, should also be achieved for a better understanding of hydrological variability and causality at all space and timescales. This could be done thanks to the future capabilities of the next generation of radar altimetry mission aiming at daily revisit, such as the SMall Altimetry Satellite for Hydrology (SMASH) constellation currently under study by the scientific community with the support of CNES.In the future, we should consider to combine the strengths of SWOT swath global measurements with a high temporal sampling of SMASH-like constellation to define a new SWOT-like satellite constellation providing global and daily to sub-daily observations.

An ongoing trend, motivated by the amount of satellite records, now covering multiple decades, along with advances in data storage and computation, has resulted in a more integrated use of satellite observations, based on synergy or data fusion techniques. A comprehensive monitoring of surface water could only benefit from integrating and merging various satellite observations with complementary strengths, such as proposed for instance in Pham-Duc et al. (2020) who combined altimetry, imagery and gravimetry observations to reveal that over the last twenty years Lake Chad is not drying.

At several points in our review, we also stressed the paucity of in situ observations in Africa. The lack of in situ observations, with a good coverage of the large spectrum of the natural variability of hydrological variable, is a limiting factor in the validation of Earth Observation products. Therefore, collection and long-term maintenance of in situ measurement networks should be supported by the agencies and service providers of satellite data, along with the development of open source-based tools to facilitate intercomparison efforts among the scientific community.

Finally, new Earth Observation techniques and methodologies are continuously being developed to help monitor surface waters and will benefit the African continent. For instance, program such the PlanetScope constellation (CubeSats, available since 2014, Cooley et al. 2019) is still underexploited and could bring new opportunities to monitor fine scale African surface water characteristics and process thanks to their capacity to provide ultra-high (3–5 m) resolution observations on a daily basis. The Global Navigation Satellite System-Reflectometry (GNSS-R) also offers new promising potentials (Chew and Small 2020) with the use of Cyclone GNSS (CYGNSS) constellation to capture surface water dynamic. Besides the concept of satellite missions, the advent of new space-borne measurements, such as real-time high-definition video for monitoring the environment, including surface water and flood for instance, or measurements from unmanned aerial vehicles (UAVs) or small unmanned drones offer great upcoming opportunities. In the near future, this will probably push back the current spatiotemporal constraints and will deliver new hydrological process insights (McCabe et al. 2017). Nevertheless, the unprecedented availability of information, with the possible production of petabytes of data will challenge the current storage and analysis capabilities. This will require new analytical approaches and capacities to interpret such massive data volumes. New tools or fusion techniques based on artificial intelligence and increased computing power will be needed. Such new opportunities will require the joint effort of space agencies, the commercial sector and start-up companies.

*Way forward* Monitoring and modeling the components of the hydrological cycle with Earth Observation have now become a reality and this will be reinforced further in the coming years. However, there is still an open path to bring all these scientific advances forward into effective applications in water resources management and related decision-making. Africa, like many regions worldwide, is currently facing an extensive human footprint on its freshwater which raises a new set of needs in this context. Complex water management problems thus call for integrated planning using Earth Observation monitoring tools. Remote sensing has the potential to emulate essential information for decision-makers (Fassoni-Andrade et al. 2021) and is bringing new ways to monitor “politically ungauged” regions where information is not publicly available as advocate by Gleason and Durand (2020). But these Earth Observation advances need to translate into real support to water and environmental governance. Thus, the remote sensing community is now facing real challenges as it needs to promote these new knowledges and datasets in a manner that is more useful for societies and to pave the way for innovation and decision support in Africa.

Potential actions to take include to reducing the gap between scientific communities, by promoting interdisciplinary approaches toward more inclusive water systems, by training decision-makers to Earth Observation current progress and advances, by promoting open access datasets, tools and repositories. These actions should also include a better integration and support of African scientists in future programs related to water in order to reduce the disparity of opportunity and access to resources. Investment in science, from national and international communities, is also needed to fill these gaps, as demonstrated for the Congo Basin (White et al. 2021).

Initiatives such as Earth Observation Science for Society from the European Space Agency (https://eo4society.esa.int/) or NASA-SERVIR, especially its declination over Africa (https://www.nasa.gov/mission_pages/servir/africa.html), are useful tools for such promotion so that remote sensing could be incorporated into applications.

Earth Observation brings also large opportunities for operational monitoring, i.e., providing observations in real time (or near real time), simulating the potential of the water resource for hydropower plants, irrigation systems, fluvial transport and freight, and we expect these capabilities to be an area of great development and application interest in the coming years. New progress is expected, especially when integrating such information into existing tools and platforms dedicated to the planning and management of water resources, such as for instance the Congo Basin Catchment Information System (https://cbcis.info/), or the Satellite-based water monitoring and flow forecasting for the Niger River Basin project (http://www.sath.abn.ne/index.html).

## Data Availability

This is a review paper for which no new data were generated. Data supporting the figures are available via the cited references.
